# Smooth muscle liver kinase B1 inhibits foam cell formation and atherosclerosis via direct phosphorylation and activation of SIRT6

**DOI:** 10.1038/s41419-023-06054-x

**Published:** 2023-08-22

**Authors:** Qiming Deng, Hongxuan Li, Xiaolin Yue, Chenghu Guo, Yuanyuan Sun, Chang Ma, Jiangang Gao, Yue Wu, Bin Du, Jianmin Yang, Cheng Zhang, Wencheng Zhang

**Affiliations:** 1 National Key Laboratory for Innovation and Transformation of Luobing Theory; The Key Laboratory of Cardiovascular Remodeling and Function Research, Chinese Ministry of Education, Chinese National Health Commission and Chinese Academy of Medical Sciences; Department of Cardiology, Qilu Hospital of Shandong University, Jinan, China; 2 School of Life Science and Key Laboratory of the Ministry of Education for Experimental Teratology, Shandong University, Jinan, China; 3 Department of Cardiology, The First Affiliated Hospital of Xi’an Jiaotong University, Xi’an, China

**Keywords:** Atherosclerosis, Atherosclerosis

## Abstract

Foam cell formation is a hallmark of the early phase of atherosclerosis. Growing evidence has demonstrated that vascular smooth muscle cells (VSMCs) comprise a considerable proportion of foam cells. Liver kinase B1 (LKB1) plays a crucial part in cardiovascular diseases. However, the role of LKB1 in VSMC-derived foam cell formation and atherosclerosis remains unclear. To explore the effects of LKB1 on VSMC-derived foam cell formation and atherosclerosis, we generated smooth muscle-specific LKB1 knockout (LKB1^SMKO^) mice by crossbreeding LKB1^flox/flox^ mice with SM22α-CreER^T2^ mice. LKB1 expression decreased in plaque-loaded aortas and oxidized low-density lipoprotein (oxLDL)-treated VSMCs. Compared with controls, atherosclerosis development was exacerbated in LKB1^SMKO^ mice via the promotion of VSMC-derived foam cell formation. Conversely, LKB1 overexpression inhibited lipid uptake and foam cell formation in VSMCs. Mechanistically, LKB1 binds to SIRT6 and directly phosphorylates and activates it, thereby reducing lectin-like oxLDL receptor-1 (LOX-1) via SIRT6-dependent histone deacetylation. Finally, adeno-associated virus (AAV)-mediated LOX-1 deficiency in smooth muscle ameliorated atherosclerosis in LKB1^SMKO^ mice. Our findings suggest that LKB1 may modulate VSMC-derived foam cell formation and atherosclerosis via the phosphorylation and activation of SIRT6.

## Introduction

As the primary underlying cause of many serious cardiovascular diseases (CVDs) such as myocardial infarction, stroke, and ischemic heart failure, atherosclerosis severely threatens human health owing to its high morbidity and mortality worldwide [1, 2]. Despite great advances in therapeutic options over the past few decades, the global burden of atherosclerotic diseases still remains [3]. Therefore, there is an urgent need to develop new therapeutic approaches to prevent atherosclerosis at an early stage. Atherosclerosis is initiated by increased plasma low density lipoprotein (LDL) particles invading the arterial wall and accumulating in the subendothelial space, where LDL particles are protected from plasma antioxidants and modified into pro-inflammatory particles, mainly oxidized LDL (oxLDL) particles [4]. In response to inflammatory factors, specific cells are recruited to engulf modified lipoproteins by multiple scavenger receptors such as CD36, scavenger receptor class A (SR-A), and lectin-like oxLDL receptor-1 (LOX-1) [5]. Cholesterol in lipid-loaded cells can be exported through reverse cholesterol transporters, including scavenger receptor class B type 1 (SR-B1), ATP binding cassette subfamily A member 1(ABCA1), and ATP binding cassette subfamily G member 1 (ABCG1) [6]. The imbalance between lipid uptake and efflux gives rise to excess intracellular lipid deposition and promotes foam cell formation, which fuels atheroma development [7, 8]. Restraining foam cell formation, which is regarded as a hallmark of the early phase of atherosclerotic lesions, may be a potential strategy for retarding disease progression [9].

Historically, macrophages that differentiated from circulating monocytes that adhere to the arterial endothelium and migrate to the subendothelial layer have been considered as the main origin of foam cells [10]. Recent studies have used immunostaining and flow cytometry to reveal that vascular smooth muscle cells (VSMCs) constitute a considerable proportion of foam cells in humans and mice [11–13]. This view was verified by pioneering researches on the application of lineage tracing [14, 15]. VSMCs constitute the main structure of the tunica media and exhibit high phenotypic plasticity and functional complexity in atherosclerosis [16, 17]. Under cholesterol loading and inflammatory stimuli, VSMCs proliferate and migrate to the intima, where some of them convert to a foam cell phenotype similar to that of macrophages and form an atheroma [14, 18]. However, the underlying mechanism of VSMC-derived foam cell formation is still unclear.

Sirtuin 6 (SIRT6) belongs to the sirtuin family of class III NAD^+^-dependent histone deacetylases and is located mainly in the nucleus, where it catalyzes deacetylation or ribosylation of various proteins, especially deacetylation of histone 3 on lysine 9 (H3K9) and lysine 56 (H3K56) [19, 20]. Previous articles demonstrated that SIRT6 could prevent some vascular diseases including atherosclerosis, pulmonary arterial hypertension, abdominal aortic aneurysm, and vascular calcification via protecting VSMCs from senescence, proliferation, phenotype transformation, and osteogenic differentiation [21–24]. This indicates the essential role of SIRT6 in the phenotypic and functional modulation of VSMCs. However, the role of SIRT6 in regulating VSMC-derived foam cell formation remains unclear.

Liver kinase B1 (LKB1), recognized as a tumor suppressor, was first identified as a loss-of-function mutation in Peutz–Jeghers syndrome [25]. LKB1 is a highly conserved and widely expressed serine/threonine kinase that is involved in the regulation of cell growth, metabolism and polarity [26]. LKB1 regulates a variety of biological processes mainly by phosphorylating and activating AMP-activated protein kinase (AMPK) family members [27]. Constitutive knockout of LKB1 has been shown to result in lethality of mouse embryos due to significant developmental abnormalities, including vascular defects [28]. Moreover, studies using mice with conditional LKB1 deletions have further indicated that LKB1 plays an important role in CVDs. Our group has previously reported that endothelial-specific LKB1 knockout mice exhibited significant endothelial dysfunction and hypertension [29]. Additionally, smooth muscle LKB1 inhibits vascular calcification and angiotensin II (Ang II)-induced abdominal aortic aneurysm formation [30, 31]. However, the role of LKB1 in VSMC-derived foam cell formation and atherosclerosis has not been elucidated.

In this study, we used smooth muscle-specific LKB1 knockout (LKB1^SMKO^) mice to investigate the function of LKB1 in atherosclerosis. Our study suggests a promising strategy for the prevention and treatment of atherosclerosis.

## Results

### LKB1 expression was decreased in atherosclerotic plaques

**Fig. 1:**
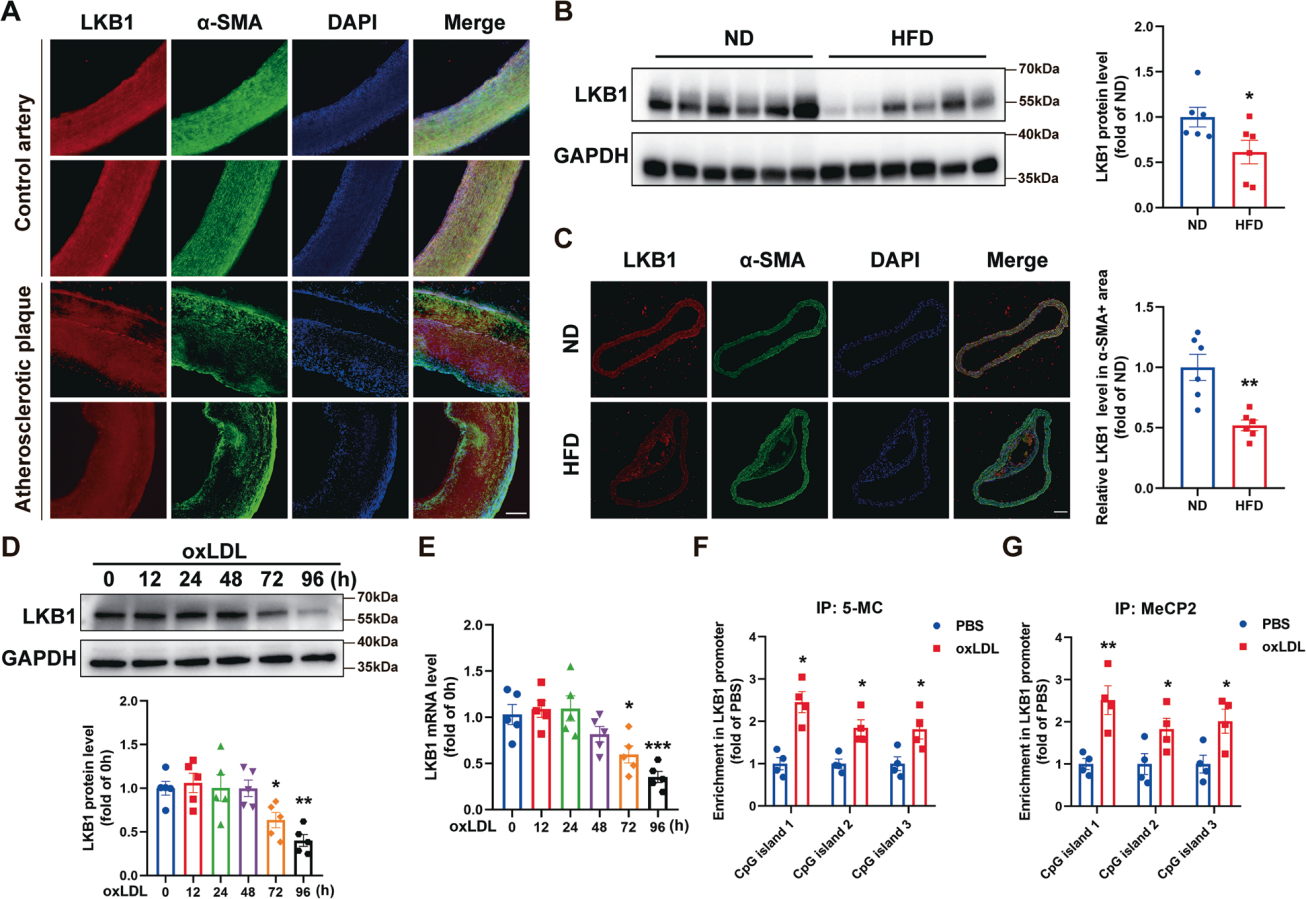
Liver kinase B1 (LKB1) expression was decreased in atherosclerotic plaques. **A** Double labeling immunofluorescent staining of LKB1 and α-SMA in control carotid arteries or carotid atherosclerotic plaques from patients who underwent carotid endarterectomy. Scale bar = 200 μm. **B** Western blot analysis of LKB1 protein level in aortas from apolipoprotein E-knockout (ApoE^−/−^) mice fed a high-fat diet (HFD) or normal diet (ND) (*n* = 6). ^*^P < 0.05 *vs* ND. **C** Double labeling immunofluorescent staining of LKB1 and α-SMA in aortas from ApoE^−/−^ mice fed a HFD or ND. Scale bar = 100 μm. ^**^P < 0.01 *vs* ND. **D** Western blot analysis of LKB1 protein level in vascular smooth muscle cells (VSMCs) treated with 100 μg/mL oxidized low-density lipoprotein (oxLDL) for different times (*n* = 5). ^*^P < 0.05, ^**^P < 0.01 *vs* 0 h. **E** Quantitative PCR (qPCR) analysis of LKB1 mRNA level in VSMCs treated with 100 μg/mL oxLDL for different times (*n* = 5). ^*^P < 0.05, ^***^P < 0.001 *vs* 0 h. **F** Methylated DNA immunoprecipitation (MeDIP) assays with anti-5-MC and qPCR analysis to determine the methylation of DNA in LKB1 promoter (*n* = 4). ^*^P < 0.05 *vs* PBS. **G** Chromatin immunoprecipitation (ChIP) assays with anti-MeCP2 and qPCR analysis to determine the methylation of DNA in LKB1 promoter (*n* = 4). ^*^P < 0.05, ^**^P < 0.01 *vs* PBS. Data were analyzed by two-tailed Student’s unpaired *t*-test (**B**, **C**, **F** and **G**) or one-way ANOVA followed by Bonferroni multiple comparison analysis (**D**, **E**).

To investigate whether LKB1 in VSMCs contributes to atherosclerosis, atherosclerotic plaques from patients who underwent carotid endarterectomy and control carotid arteries from normal donors were obtained and examined by immunofluorescence. As compared with control arteries, LKB1 expression was decreased in atherosclerotic plaques (Fig. 1A). ApoE^−/−^ mice were fed normal diet (ND) or high-fat diet (HFD) for 12 weeks. When compared with the ND group, LKB1 protein level was significantly reduced in the aortas of mice in the HFD group (Fig. 1B). Additionally, we used immunofluorescence to evaluate LKB1 expression in VSMCs that labeled by α-SMA and found that LKB1 level was decreased in aortic smooth muscle from the HFD group as compared with the ND group (Fig. 1C). The VSMCs extracted from the aortas of mice were used for the following in vitro experiments. LKB1 protein levels decreased following in vitro oxLDL (100 μg/mL) treatment after 72 h (Fig. 1D). Moreover, the mRNA level of LKB1 began to decrease after 72 h of oxLDL treatment (Fig. 1E).

Next, we determined whether oxLDL downregulated LKB1 transcription through promoter methylation. VSMCs were treated with oxLDL for 72 h, followed by methylated DNA immunoprecipitation (MeDIP) assays with anti-5-MC antibody (for recognizing methylated DNA) and chromatin immunoprecipitation (ChIP) assays with anti-MeCP2 antibody (for recognizing the specific protein bound to methylated DNA). PCR results with chromatin DNA from ChIP or MeDIP assays and primers targeting CpG islands in the LKB1 promoter showed that the LKB1 promoter of oxLDL-treated VSMCs was more methylated than that of the control (Fig. 1F, G), suggesting that oxLDL downregulated LKB1 expression via promoter DNA methylation in VSMCs.

### LKB1 deficiency in smooth muscle exacerbated atherosclerosis

**Fig. 2:**
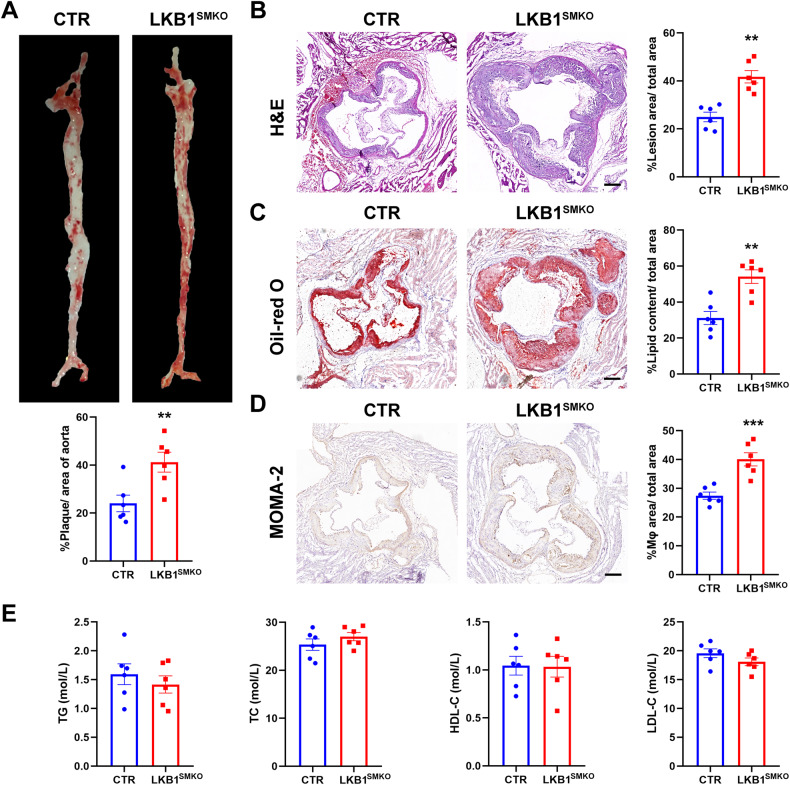
LKB1 deficiency in smooth muscle exacerbated atherosclerosis. Control (CTR) and smooth muscle-specific LKB1 knockout (LKB1^SMKO^) mice were injected with rAAV8/D377Y-mPCSK9 and fed a Paigen diet for 12 weeks. **A** Oil-red O staining in aortas (*n* = 6). ^**^P < 0.01 *vs* CTR. **B** Hematoxylin and eosin (H&E) staining in aortic roots (*n* = 6). Scale bar = 200 μm. ^**^P < 0.01 *vs* CTR. **C** Oil-red O staining in aortic roots (*n* = 6). Scale bar = 200 μm. ^**^P < 0.01 *vs* CTR. **D** Immunohistochemical staining of MOMA-2 in aortic roots (*n* = 6). Scale bar = 200 μm. ^***^P < 0.001 *vs* CTR. **E** Levels of total cholesterol (TC), triglycerides (TG), high-density lipoprotein cholesterol (HDL-C), and low-density lipoprotein cholesterol (LDL-C) in serum from CTR and LKB1^SMKO^ mice (*n* = 6). Data were analyzed by two-tailed Student’s unpaired *t*-test (**A**–**E**).

To explore the role of smooth muscle LKB1 in atherosclerosis, an atherosclerotic animal model was established using control (CTR) and LKB1^SMKO^ mice injected with AAV8/D377Y-mPCSK9 and fed a Paigen diet for 12 weeks. The atherosclerotic lesion areas for aortas *en face* and cross-sectional aortic roots were both larger in LKB1^SMKO^ mice than CTR (41.22 ± 4.16% vs 21.02 ± 3.44%, *P* < 0.01; 41.73 ± 2.53% vs 24.96 ± 1.94%, *P* < 0.001) (Fig. 2A, B). Lipid burden measured by Oil-red O-stained aortic roots showed a significant increase in the area of the lesion in LKB1^SMKO^ mice than in control mice (54.12 ± 3.75% vs 31.15 ± 3.65%, *P* < 0.01) (Fig. 2C). Furthermore, LKB1 deletion increased macrophage accumulation in the plaques, as determined by immunostaining with an anti-MOMA-2 antibody (Fig. 2D). However, LKB1 knockout in the smooth muscle did not affect the serum levels of triglycerides (TG), total cholesterol (TC), high-density lipoprotein cholesterol (HDL-C), or low-density lipoprotein cholesterol (LDL-C) (Fig. 2E). Thus, smooth muscle-specific LKB1 knockout exacerbates the development of atherosclerosis.

### LKB1 inhibited VSMC-derived foam cells formation

**Fig. 3:**
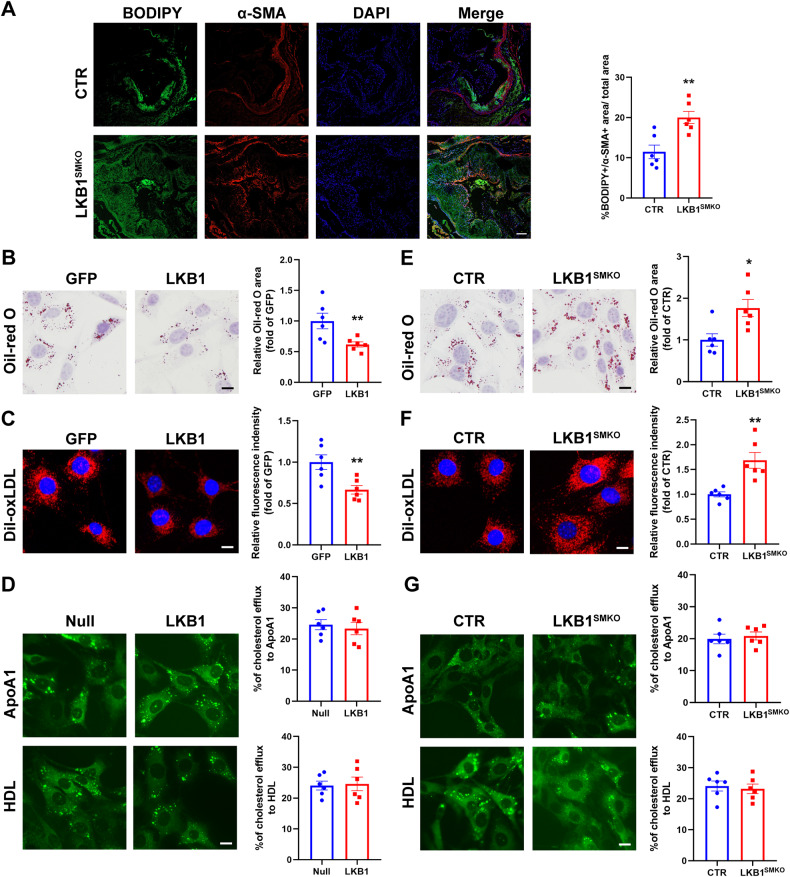
LKB1 inhibited VSMC-derived foam cell formation. **A** Double labeling immunofluorescent staining of BODIPY and α-SMA in aortic roots from CTR or LKB1^SMKO^ mice (*n* = 6). Scale bar = 100 μm. ^**^P < 0.01 *vs* CTR. **B** VSMCs were infected with adenovirus-expressing green fluorescent protein (GFP) or LKB1 before 24-h stimulation of oxLDL (100 μg/mL), and then subjected to Oil-red O staining (*n* = 6). Scale bar = 10 μm. ^**^P < 0.01 *vs* GFP. **C** VSMCs were infected with adenovirus-expressing GFP or LKB1 and then incubated with Dil-oxLDL (10 μg/mL) for 4 h (*n* = 6). Scale bar = 10 μm. ^**^P < 0.01 *vs* GFP. **D** VSMCs were infected with LKB1-overexpressing adenovirus or Null adenovirus before 4-h incubation of NBD-cholesterol (1 μg/mL), followed by cholesterol efflux assay. Scale bar = 10 μm. **E** VSMCs from CTR or LKB1^SMKO^ mice were treated with oxLDL for 24 h, and then subjected to Oil-red O staining (*n* = 6). Scale bar = 10 μm. ^*^P < 0.05 *vs* CTR. **F** VSMCs from CTR or LKB1^SMKO^ mice were incubated with Dil-oxLDL for 4 h (*n* = 6). Scale bar = 10 μm. ^**^P < 0.01 *vs* CTR. **G** VSMCs from CTR or LKB1^SMKO^ mice were incubated with NBD-cholesterol, followed by a cholesterol efflux assay. Scale bar = 10 μm. Data were analyzed by two-tailed Student’s unpaired t-test (**A**–**G**).

Foam cell formation plays a crucial role in the pathogenesis of atherosclerosis. To explore whether LKB1 plays a role in VSMC-derived foam cell formation, we used BODIPY to detect lipid droplets of foam cells in plaques. Cells that were positive for both BODIPY and α-SMA in plaques were considered VSMC-derived foam cells. As compared with CTR, the BODIPY + /α-SMA+ areas in plaques of LKB1^SMKO^ mice were significantly increased (Fig. 3A), which suggested that LKB1 deletion promoted VSMC-derived foam cell formation. To confirm this result, VSMCs were stimulated with oxLDL for 24 h to mimic VSMC-derived foam cells in vitro. We used adenovirus to overexpress LKB1 before 24-h stimulation with oxLDL and determined the lipid burden of VSMCs by Oil-red O staining. The results showed that compared to the GFP group, the overexpression of LKB1 significantly decreased the accumulation of lipid droplets in VSMCs (Fig. 3B). Since the intracellular lipid content depends on the balance between lipid uptake and cholesterol efflux, we investigated whether LKB1 suppresses the phagocytosis of oxLDL or enhances the efflux of cholesterol. To evaluate the capacity of VSMCs for lipid uptake, they were infected with adenovirus-expressing GFP or LKB1 and then treated with Dil-oxLDL. LKB1 overexpression inhibited Dil-oxLDL uptake by VSMCs (Fig. 3C). Cholesterol efflux assays showed that LKB1 did not affect cholesterol egress from the VSMCs (Fig. 3D). Primary VSMCs from CTR and LKB1^SMKO^ mice were cultured and treated with oxLDL or Dil-oxLDL. As expected, lipid deposition and lipid uptake were increased by LKB1 deficiency (Fig. 3E, F). Additionally, LKB1 deficiency did not affect cholesterol efflux (Fig. 3G). Thus, we concluded that LKB1 could inhibit VSMC-derived foam cell formation.

### LKB1 inhibits the expression of oxLDL receptor LOX-1

**Fig. 4:**
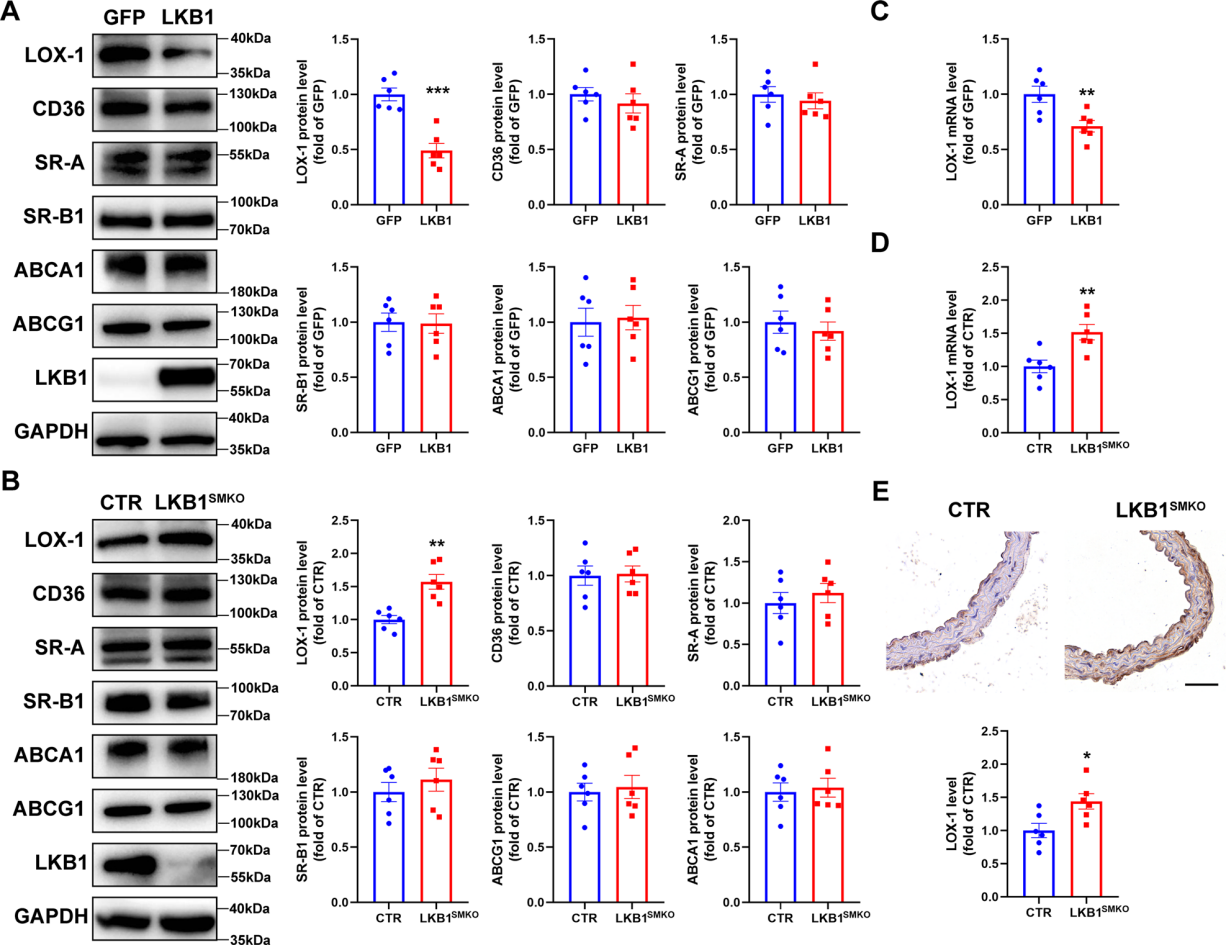
LKB1 inhibits the expression of lectin-like oxidized low-density lipoprotein receptor-1 (LOX-1). **A** Western blot analysis of cholesterol uptake and reverse cholesterol transport proteins in VSMCs infected with adenovirus-expressing GFP or LKB1 (*n* = 6). ^***^P < 0.001 *vs* GFP. **B** Western blot analysis of cholesterol uptake and reverse cholesterol transport proteins in VSMCs from CTR or LKB1^SMKO^ mice (*n* = 6). ^**^P < 0.01 *vs* CTR. **C** QPCR analysis of LOX-1 mRNA in VSMCs (*n* = 6). ^**^P < 0.01 *vs* GFP. **D** QPCR analysis of LOX-1 mRNA in VSMCs (*n* = 6). ^**^P < 0.01 *vs* CTR. **E** Immunohistochemistry staining of LOX-1 in aortas from CTR or LKB1^SMKO^ mice (*n* = 6). Scale bar = 100 μm. ^*^P < 0.05 *vs* CTR. Data were analyzed by two-tailed Student’s unpaired *t*-test (**A**–**E**).

Next, we determined the expression of proteins responsible for cholesterol uptake and reverse transport by western blotting, which showed that the protein level of LOX-1 was reduced by LKB1 overexpression and elevated by LKB1 deletion. However, LKB1 did not alter the protein levels of other scavenger receptors or reverse cholesterol transporters (Fig. 4A, B). Additionally, LKB1 overexpression decreased LOX-1 mRNA levels (Fig. 4C), whereas LKB1 deficiency increased LOX-1 mRNA levels (Fig. 4D). Furthermore, LOX-1 expression in the aorta was detected using immunohistochemical staining. Compared to CTR mice, LOX-1 expression levels in the aortas of LKB1^SMKO^ mice were significantly increased (Fig. 4E). Thus, LKB1 inhibits the expression of LOX-1, which may have contributed to the suppression of VSMC-derived foam cell formation.

### LKB1 reduces oxLDL uptake via LOX-1

**Fig. 5:**
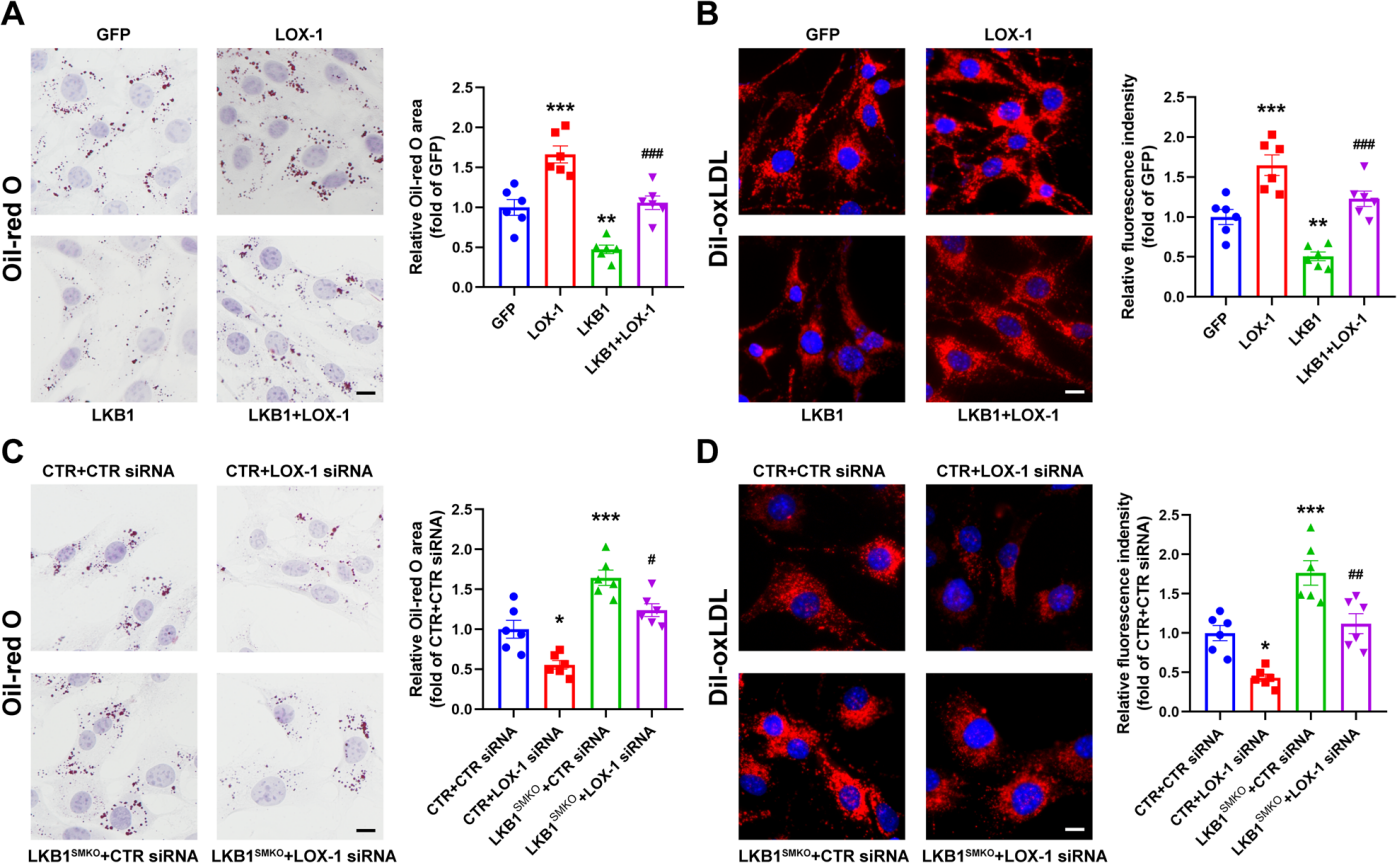
LKB1 regulates oxLDL uptake via LOX-1. **A** VSMCs were infected with adenovirus-expressing GFP, LKB1, or LOX-1, followed by oxLDL treatment and Oil-red O staining (*n* = 6). Scale bar = 10 μm. ^**^P < 0.01, ^***^P < 0.001 *vs* GFP, ^###^*P* < 0.001 *vs* LKB1. **B** VSMCs were infected with adenovirus-expressing GFP, LKB1 or LOX-1, and then incubated with Dil-oxLDL (10 μg/mL) for 4 h (*n* = 6). Scale bar = 10 μm. ^**^P < 0.01, ^***^P < 0.001 *vs* GFP, ^###^*P* < 0.001 *vs* LKB1. **C** VSMCs from CTR or LKB1^SMKO^ mice were transfected with CTR siRNA or LOX-1 siRNA. After 24-h stimulation of oxLDL, VSMCs were subjected to Oil-red O staining (*n* = 6). Scale bar = 10 μm. ^*^P < 0.05, ^***^P < 0.001 *vs* CTR + CTR siRNA, ^#^*P* < 0.05 *vs* LKB1^SMKO^ + CTR siRNA. **D** VSMCs from CTR or LKB1^SMKO^ mice were transfected with CTR siRNA or LOX-1 siRNA, followed by Dil-oxLDL treatment for 4 h (*n* = 6). Scale bar = 10 μm. ^*^P < 0.05, ^***^P < 0.001 *vs* CTR + CTR siRNA, ^##^*P* < 0.01 *vs* LKB1^SMKO^ + CTR siRNA. Data were analyzed by one-way ANOVA followed by Bonferroni multiple comparison analysis (**A**–**D**).

Based on these data, we hypothesized that LKB1 inhibits VSMC-derived foam cell formation by downregulating LOX-1. To test this hypothesis, VSMCs were infected with adenoviruses expressing GFP, LOX-1, or LKB1, followed by oxLDL treatment. Oil-red O staining indicated that the reduction in lipid deposition and oxLDL uptake after LKB1 overexpression was reversed by LOX-1 overexpression (Fig. 5A, B). CTR- and LKB1-deficient VSMCs were transfected with LOX-1 siRNA before incubation with oxLDL. LOX-1 deficiency consistently suppressed the enhanced lipid accumulation and uptake after LKB1 deletion (Fig. 5C, D). Collectively, these results indicate that LKB1 reduces oxLDL uptake via LOX-1.

### LKB1 regulates LOX-1 expression via SIRT6

**Fig. 6:**
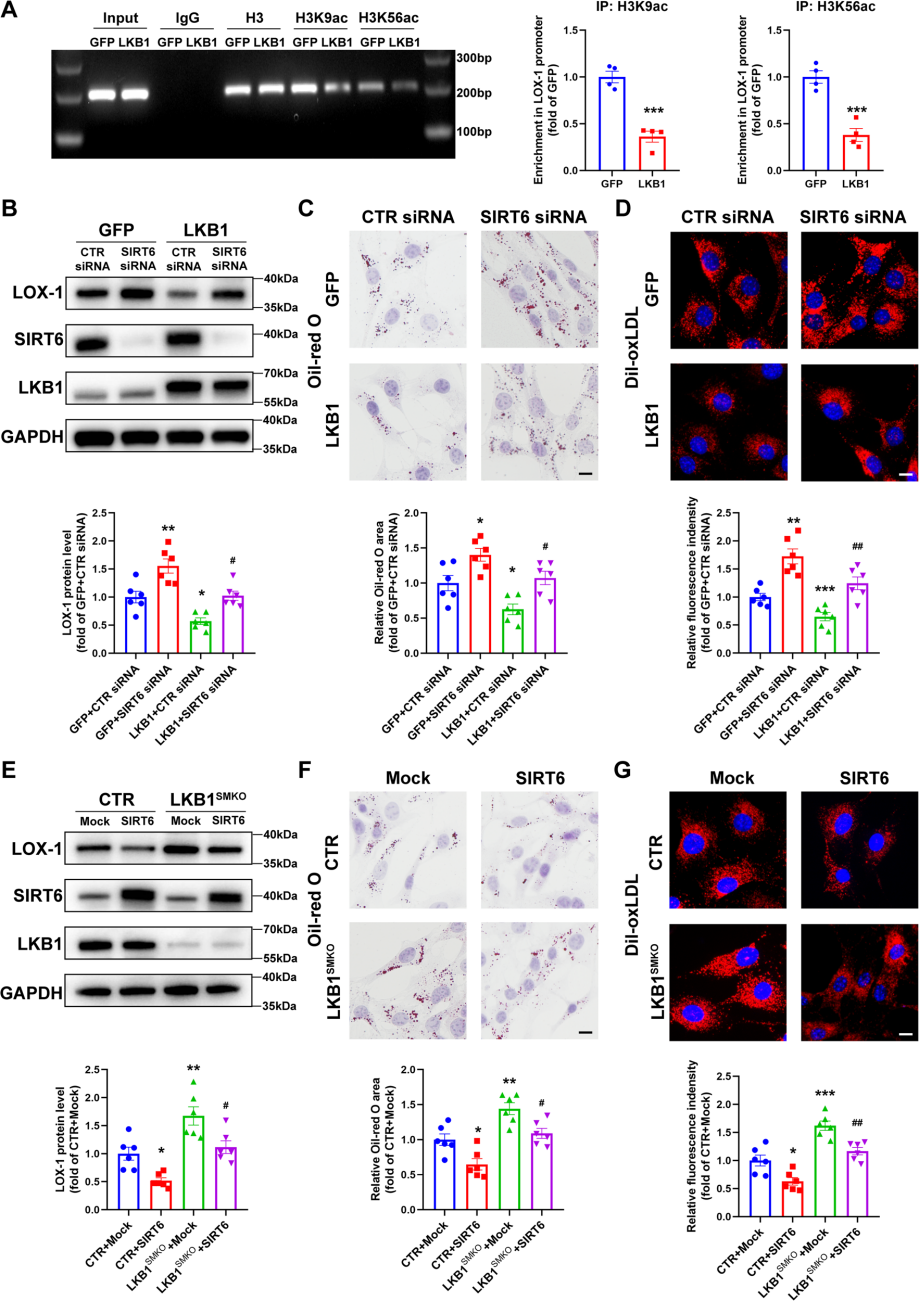
LKB1 regulates LOX-1 expression via SIRT6. **A** ChIP-PCR analysis to determine the histone acetylation of LOX-1 promoter in VSMCs infected with adenovirus-expressing GFP or LKB1 (*n* = 4). ^***^P < 0.001 *vs* GFP. **B**–**D** VSMCs were transfected with CTR siRNA or SIRT6 siRNA before infection with adenovirus-expressing GFP or LKB1. **B** Western blot analysis of LOX-1 protein (*n* = 6). ^*^P < 0.05, ^**^P < 0.01 *vs* GFP + CTR siRNA, ^#^*P* < 0.05 *vs* LKB1 + CTR siRNA. **C** Oil-red O staining after 24-h stimulation of oxLDL (*n* = 6). Scale bar = 10 μm. ^*^P < 0.05 *vs* GFP + CTR siRNA, ^#^*P* < 0.05 *vs* LKB1 + CTR siRNA. **D** Representative fluorescence images of VSMCs obtained after 4-h incubation with Dil-oxLDL. Scale bar = 10 μm. ^**^P < 0.01, ^***^P < 0.001 *vs* GFP + CTR siRNA, ^##^*P* < 0.01 *vs* LKB1 + CTR siRNA. **E**–**G** VSMCs from CTR or LKB1^SMKO^ mice were transfected with plasmids encoding the murine SIRT6 gene. **E** Western blot analysis of LOX-1 protein (*n* = 6). ^*^P < 0.05, ^**^P < 0.01 *vs* CTR+Mock, ^#^*P* < 0.05 *vs* LKB1^SMKO^+Mock. **F** Oil-red O staining after 24-h stimulation of oxLDL (*n* = 6). Scale bar = 10 μm. ^*^P < 0.05, ^**^P < 0.01 *vs* CTR+Mock, ^#^*P* < 0.05 *vs* LKB1^SMKO^+Mock. **G** Representative fluorescence images of VSMCs obtained 4-h incubation with Dil-oxLDL (*n* = 6). Scale bar = 10 μm. ^*^P < 0.05, ^***^P < 0.001 *vs* CTR+Mock, ^##^*P* < 0.01 *vs* LKB1^SMKO^+Mock. Data were analyzed by two-tailed Student’s unpaired *t*-test (**A**) or one-way ANOVA followed by Bonferroni multiple comparison analysis (**B**–**G**).

To investigate the molecular mechanism by which LKB1 regulates LOX-1 expression, Compound C was used to block the canonical LKB1-AMPK signaling pathway. We found that AMPK inhibition did not abolish the effect of LKB1 on the downregulation of LOX-1 (Fig. S1), suggesting that LKB1 regulated LOX-1 expression in an AMPK-independent manner. To determine whether histone acetylation participates in the regulatory effect of LKB1 on LOX-1 expression, we conducted ChIP assays for acetyl H3K9 and H3K56 in VSMCs infected with adenovirus-expressing GFP or LKB1. As shown in Fig. 6A, the levels of H3K9 and H3K56 acetylation in the LOX-1 promoter were significantly reduced upon LKB1 overexpression. SIRT6 is a nuclear deacetylase that could modulate the formation of macrophage-derived foam cells [32, 33]. To determine whether SIRT6 is involved in the regulation of LOX-1 by LKB1, GFP- or LKB1-overexpressing VSMCs were transfected with SIRT6 siRNA. SIRT6 deficiency attenuated the reduction in LOX-1 expression caused by LKB1 overexpression (Fig. 6B). Additionally, the decrease in lipid accumulation and uptake caused by LKB1 overexpression was suppressed by SIRT6 deficiency (Fig. 6C, D). Conversely, SIRT6 overexpression repressed the increase in LOX-1 expression, lipid accumulation, and lipid uptake in LKB1-deficient VSMCs (Fig. 6E–G). These data indicated that LKB1 regulates LOX-1 expression via SIRT6-mediated histone deacetylation.

### LKB1 phosphorylates and activates SIRT6

**Fig. 7:**
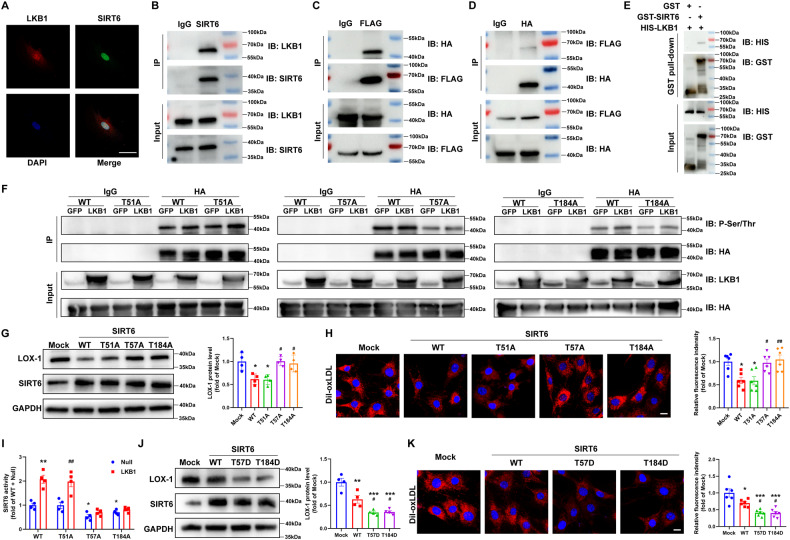
LKB1 phosphorylates and activates SIRT6. **A** Representative immunofluorescence images of co-localization of LKB1 and SIRT6 in VSMCs. Scale bar = 50 μm. **B** VSMC lysates were immunoprecipitated with IgG or anti-SIRT6. Results were determined via western blot analysis with anti-LKB1 and anti-SIRT6 antibodies. **C,** **D** HEK 293 T cells were transfected with plasmids encoding Flag-tagged LKB1 and HA-tagged SIRT6, and lysates were immunoprecipitated with IgG and anti-Flag (**C**) or anti-HA (**D**) antibodies respectively. Results were determined via western blot analysis with anti-HA and anti-Flag antibodies. **E** Purified His-LKB1 proteins were incubated with GST or GST-SIRT6-conjugated GSH beads, the boiled eluates were then separated and detected by western blot analysis with anti-His and anti-GST antibodies. **F** HEK 293 T cells were transfected with plasmids encoding HA-SIRT6-WT/T51A/T57A/T184A and adenovirus expressing GFP or LKB1, lysates were immunoprecipitated with IgG or anti-HA. Results were determined via western blot analysis with anti-P-Ser/Thr and anti-HA antibodies. **G**, **H** VSMCs were transfected with plasmids encoding HA-SIRT6-WT/T51A/T57A/T184A. **G** Western blot analysis of LOX-1 protein (*n* = 4). ^*^P < 0.05 *vs* Mock, ^#^*P* < 0.05 *vs* WT. **H** Representative fluorescence images of VSMCs obtained after 4-h incubation with Dil-oxLDL (*n* = 6). Scale bar = 10 μm. ^*^P < 0.05 *vs* Mock, ^#^*P* < 0.05, ^##^*P* < 0.01 *vs* WT. **I** SIRT6 activity measurement of nuclear extract from VSMCs transfected with plasmids encoding HA-SIRT6-WT/T51A/T57A/T184A and LKB1-overexpressing adenovirus or null adenovirus (*n* = 4). ^**^P < 0.01, ^***^P < 0.001 *vs* WT+Null, ^#^*P* < 0.05 *vs* T51A+Null. **J**, **K** VSMCs were transfected with plasmids encoding HA-SIRT6-WT/T57D/T184D. **J** Western blot analysis of LOX-1 protein (*n* = 4). ^*^P < 0.05, ^***^P < 0.001 *vs* Mock, ^#^*P* < 0.05 *vs* WT. **K** Representative fluorescence images of VSMCs obtained after 4-h incubation with Dil-oxLDL (*n* = 6). Scale bar = 10 μm. ^*^P < 0.05, ^***^P < 0.001 *vs* Mock, ^#^*P* < 0.05 *vs* WT. Data were analyzed by one-way ANOVA followed by Bonferroni multiple comparison analysis (**G**–**K**).

To verify the interactions between LKB1 and SIRT6, we performed immunofluorescence staining with antibodies against these two proteins, which showed partial colocalization of LKB1 and SIRT6 in VSMCs (Fig. 7A). Immunoprecipitation assays revealed that LKB1 could bind to SIRT6 in VSMCs (Fig. 7B). This was further confirmed in HEK 293 T cells which were transfected with Flag-tagged LKB1 and HA-tagged SIRT6 (Fig. 7C, D). Furthermore, the GST pull-down assay demonstrated that LKB1 could bind to SIRT6 directly (Fig. 7E).

Next, we investigated the effects of LKB1 on SIRT6 expression. Surprisingly, LKB1 overexpression or knockdown did not affect SIRT6 expression (Fig. S2A, B). LKB1 is a serine/threonine kinase that phosphorylates and activates downstream proteins. We used Group-based Prediction System (GPS, version 5.0) software to predict the possible LKB1 phosphorylation sites of SIRT6. The human, rat, and mouse SIRT6 sequences showed three conserved LKB1 phosphorylation sites (threonines 51, 57, and 184) in the catalytic domain of sirtuin (Table S2). To examine whether LKB1 could phosphorylate SIRT6 at the predicted sites, plasmids carrying the phosphorylation site mutant SIRT6 (threonine 51, 57, and 184 replaced with alanine) were constructed. HEK 293 T cells were transfected with plasmids carrying HA-SIRT6-WT or three different mutants (HA-SIRT6-T51A, T57A, and T184A) and then infected with GFP- or LKB1-overexpressing adenovirus. Coimmunoprecipitation with anti-HA or IgG was performed, followed by western blot analysis using anti-phospho-(Ser/Thr) or anti-HA primary antibodies. The results indicated that LKB1 elevated the phosphorylation levels of HA-SIRT6-WT and HA-SIRT6-T51A, but not those of HA-SIRT6-T57A or HA-SIRT6-T184A (Fig. 7F). Additionally, HA-SIRT6-WT and HA-SIRT6-T51A significantly decreased LOX-1 expression and lipid uptake by VSMCs, whereas these factors did not differ with HA-SIRT6-T57A and HA-SIRT6-T184A (Fig. 7G, H). Additionally, LKB1 significantly increased the deacetylase activity of SIRT6 as measured by the SIRT6 Activity Assay Kit in VSMCs, while mutations in alanine at threonine 57 or 184 blocked SIRT6 activation by LKB1 (Fig. 7I). Additionally, SIRT6 expressed by phosphomimetic mutants (threonine was mutated to aspartic acid, HA-SIRT6-T57D, and HA-SIRT6-T184D) further decreased LOX-1 expression and lipid uptake of VSMCs compared to HA-SIRT6-WT (Fig. 7J, K). Taken together, these data demonstrate that LKB1 phosphorylates SIRT6 at Thr 57 and 184 sites, which activates SIRT6, resulting in decreased LOX-1 expression and lipid uptake in VSMCs.

### Smooth muscle LOX-1 deficiency ameliorates atherosclerosis in LKB1^SMKO^ mice

**Fig. 8:**
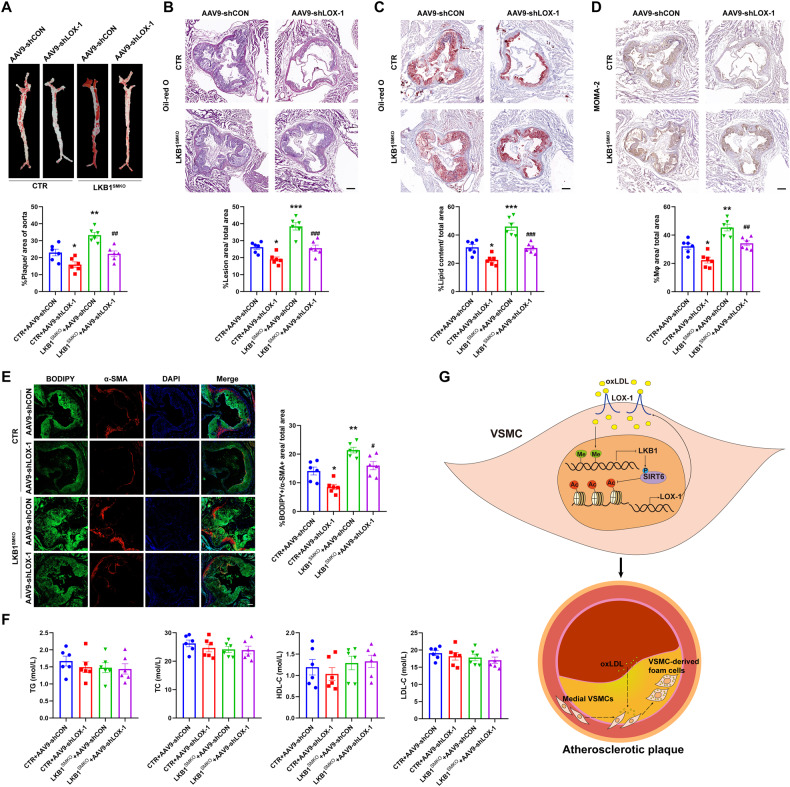
Smooth muscle LOX-1 deficiency ameliorates atherosclerosis in LKB1^SMKO^ mice. CTR and LKB1^SMKO^ mice were injected with AAV9-shCON or AAV9-shLOX-1 1 week before injection with rAAV8/D377Y-mPCSK9 and a Paigen diet feeding for 12 weeks. **A** Oil-red O staining in aortas (*n* = 6). ^*^P < 0.05, ^**^P < 0.01 *vs* CTR + AAV9-shCON, ^##^*P* < 0.01 *vs* LKB1^SMKO^ + AAV9-shCON. **B** Hematoxylin and eosin (H&E) staining in aortic roots (*n* = 6). Scale bar = 200 μm. ^*^P < 0.05, ^***^P < 0.001 *vs* CTR + AAV9-shCON, ^###^*P* < 0.001 *vs* LKB1^SMKO^ + AAV9-shCON. **C** Oil-red O staining in aortic roots (*n* =6). Scale bar = 200 μm. ^*^P < 0.05, ^***^P < 0.001 *vs* CTR + AAV9-shCON, ^###^*P* < 0.001 *vs* LKB1^SMKO+^AAV9-shCON. **D** Immunohistochemical staining of MOMA-2 in aortic roots (*n* = 6). Scale bar = 200 μm. ^*^P < 0.05, ^**^P < 0.01 *vs* CTR + AAV9-shCON, ^##^*P* < 0.01 *vs* LKB1^SMKO^ + AAV9-shCON. **E** Double labeling immunofluorescent staining of BODIPY and α-SMA in aortic roots (*n* = 6). Scale bar = 100 μm. ^*^P < 0.05, ^**^P < 0.01 *vs* CTR + AAV9-shCON, ^#^*P* < 0.05 *vs* LKB1^SMKO^ + AAV9-shCON. **F** Levels of TC, TG, HDL-C, and LDL-C in serum. Data were analyzed by one-way ANOVA followed by Bonferroni multiple comparison analysis (**A**–**F**). **G** Smooth muscle LKB1 inhibits VSMC-derived foam cell formation and atherosclerosis via phosphorylation of SIRT6 and subsequent inhibition of LOX-1 expression.

To explore whether smooth muscle LKB1 deficiency facilitates atherosclerosis via LOX-1 in vivo, CTR and LKB1^SMKO^ mice were infected with AAV9-shCON or AAV9-shLOX-1 under SM22α promoter, injected with AAV8/D377Y-mPCSK9, and fed a Paigen diet for 12 weeks. Compared to CTR, LKB1^SMKO^ mice displayed a heavier plaque load, which was attenuated by AAV9-shLOX-1 (Fig. 8A–D). Furthermore, the increase of BODIPY + /α-SMA+ areas in lesions due to smooth muscle LKB1 deletion was also suppressed by AAV9-shLOX-1 (Fig. 8E). However, smooth muscle LOX-1 deficiency did not affect the levels of TG, TC, HDL-C, or LDL-C in LKB1^SMKO^ mice (Fig. 8F). Therefore, atherosclerosis aggravated by smooth muscle LKB1 deletion was ameliorated by LOX-1 deficiency in vivo.

## Discussion

In this study, we used smooth muscle-specific LKB1 knockout mice to explore the role of smooth muscle LKB1 in the development of atherosclerosis in vivo and found that LKB1 deletion in smooth muscle exaggerated atherosclerosis in mice. In vitro, LKB1 overexpression inhibited VSMC-derived foam cell formation, whereas LKB1 deficiency facilitated lipid accumulation in VSMCs. Mechanistically, LKB1 binds to SIRT6 and activates it via phosphorylation, thereby reducing the expression of LOX-1 via SIRT6-dependent histone deacetylation.

LKB1, also known as serine-threonine kinase 11 (STK11), has been identified as a tumor suppressor in various cancers, including gastrointestinal, non-small cell lung, pancreatic, cervical, and myeloproliferative neoplasms [34, 35]. Mounting evidence suggests that LKB1 is involved in maintaining cellular phenotype during the development of non-neoplastic diseases including CVDs [36]. Given that macrophage LKB1 ameliorates atherosclerosis [37], we measured the expression levels of LKB1 in the aortas of plaque-loaded mice and VSMCs treated with oxLDL to determine whether smooth muscle LKB1 participates in atherosclerosis. As shown in this study, smooth muscle LKB1 expression decreased both in vivo and in vitro under pathological stimuli. A wide array of studies has confirmed the involvement of epigenetic reprogramming in the pathogenesis of atherosclerosis [38–40]. DNA methylation is the most widely researched epigenetic mechanism, and oxLDL has been reported to alter DNA methylation in promoters of atherosclerosis-related genes in endothelial cells and leukocytes [41, 42]. A recent study indicated that exposure to cigarette smoke extract downregulated LKB1 transcription via CpG island methylation in non-small cell lung cancer cells [43]. In this study, we found that oxLDL downregulates LKB1 expression via promoter DNA methylation.

Foam cell formation is a characteristic event that occurs during the early stages of atherosclerosis. Lipid accumulation promotes the differentiation of VSMCs and formation of VSMC-derived foam cells in the arterial wall [44]. Herein, smooth muscle-specific LKB1 knockout mice exhibited exacerbated atherosclerosis with more α-SMA positive foam cells in plaques. This suggested that LKB1 deficiency facilitated VSMC-derived foam cell formation. It has been reported that oxLDL could also induce foam cell formation [45]. In this study, primary VSMCs treated with oxLDL or Dil-oxLDL further showed that LKB1 affected VSMC-derived foam cell formation by modulating lipid uptake.

Numerous studies have confirmed that LKB1 plays a vital role in metabolic regulation. In addition to glucose homeostasis [46, 47], LKB1 also takes part in lipid metabolism. LKB1 deficiency can accelerate nonalcoholic fatty liver disease and chronic kidney disease due to lipid metabolic disorders [48, 49]. In this study, we determined the expression levels of proteins mediating cholesterol uptake or efflux to research how LKB1 modulates the lipid metabolism in VSMCs. We found that smooth muscle LKB1 deletion significantly increased the expression of LOX-1, rather than SR-A which was reported to be downregulated by LKB1 in macrophages [37]. A recent study demonstrated that LOX-1, but not CD36 or SR-A, was significantly elevated by oxLDL in VSMCs, and that LOX-1 silencing decreased intracellular lipid deposition and inhibited VSMC-derived foam cell formation [50], which in accordance with the results of our study. The human LOX-1 is encoded by *oxLDL receptor-1* (*OLR1*), which is located in the C-type lectin gene cluster of chromosome 12 [51]. Transcription factor NF-κB and AP-1 could bind at the 5’-regulatory regions of the LOX-1 gene [52]. The binding of oxLDL to LOX-1 activates NF-κB and enhances the transcription of LOX-1 gene [53]. Additionally, several studies have shown that various miRNAs, such as miR-155, miR-590-5P, and let-7, regulate LOX-1 expression, mainly in a reciprocal fashion [54–56]. In this study, we found that LKB1 significantly reduced the acetylation levels of specific histone deacetylation sites (H3K9 and H3K56) for SIRT6 in the LOX-1 promoter, which provides a new regulatory pathway for LOX-1 expression and improves our understanding of the role of LKB1 in CVDs.

SIRT6 is a member of the sirtuin family of proteins, which act as deacetylases [57]. It has been discovered that SIRT6 could inhibit the formation of macrophage-derived foam cells; however, the mechanism underlying its inhibitory effect remains controversial. He et al. reported that SIRT6 overexpression elevated cholesterol efflux through upregulation of ABCA1 and ABCG1, and promoted autophagy flux by upregulating LC3B-II and ATG5 in THP-1 cells [33]. On the other hand, the findings of Arsiwala *et al*. demonstrated that SIRT6 knockdown in macrophages enhanced oxLDL uptake by increasing the level of SR-A expression in vitro and in vivo [32]. However, whether SIRT6 affects VSMC-derived foam cell formation has not been investigated before. Previous studies have shown that the expression of SIRT6 was reduced in human and mouse VSMCs in plaques, and smooth muscle-specific overexpression of SIRT6 attenuated the development of atherosclerosis [21]. Our study suggested that enhancing the deacetylase activity of SIRT6 by LKB1 could inhibit oxLDL uptake and foam cell formation through decreasing LOX-1 expression in VSMCs. At present, most studies of the SIRT6 activity regulation have focused on small molecule compounds such as resveratrol, SRT2104 and NAD precursors [58]. However, few studies have focused on SIRT6 phosphorylation and activation. Serine 338 (S338) is the most reported phosphorylation site on SIRT6 [59–61]. AKT1 phosphorylates SIRT6 at S338 and enhances the degradation of SIRT6 through the ubiquitin-proteasome pathway in breast cancer cells [59]. In this study, we found that LKB1 could bind and phosphorylate SIRT6 at Thr 57 and 184 sites resulting in its activation. Our study provides a new modulation pattern of SIRT6 activity.

Based on previous clinical researches, the genes involved in this study, including *STK11*, *SIRT6*, and *OLR1*, have exhibited significant relevance to the risk of CVDs. Moreover, specific single nucleotide polymorphisms (SNPs) within these genes have been identified in association with atherosclerotic diseases. For the *STK11* gene, genetic variability at rs12977689 has been linked to an increased risk of coronary artery disease (CAD) in type 2 diabetes patients [62]. In the case of SIRT6, the reduced serum concentration of SIRT6 independently correlated with the diagnosis of CAD [63] and several SNPs within the *SIRT6* gene were found to be associated with the susceptibility or severity of CAD [64, 65]. As for *OLR1* gene, a variation at rs11053646 has been shown to increase the risk of hypertension, myocardial infarction, carotid atherosclerosis, and ischemic stroke [66–69]. The collective clinical evidence underscores the vital roles of LKB1, SIRT6, and LOX-1 in atherosclerotic diseases and suggests their potential as prospective targets for clinical interventions.

In conclusion, we revealed that smooth muscle LKB1 inhibits VSMC-derived foam cell formation and atherosclerosis via direct phosphorylation and activation of SIRT6 and subsequent inhibition of LOX-1 expression. Our findings clarify the mechanism of VSMC-derived foam cell formation and provide a promising strategy for the prevention and treatment of atherosclerosis.

## Materials and methods

### Reagents

Adenovirus expressing LKB1, LOX-1 or GFP and adenovirus vector containing no transgene expression cassette (Null) was purchased from HanBio Technology (Shanghai, China). Recombinant adeno-associated virus serotype 8 of murine proprotein convertase subtilisin/kexin type 9 mutants (AAV8/D377Y-mPCSK9) were from Vigenebio (Maryland, United States). Adeno-associated virus serotype 9 that delivers SMC-specific SM22α promoter driving control shRNA (AAV9-shCON) or shRNA against murine LOX-1(AAV9-shLOX-1) were from Vigenebio (Maryland, United States). High-fat diet (TP28521) which containing 40% fat and 1.25% cholesterol, and Paigen diet (TP28640) which containing 15% fat, 0.5% bile salt and 1.25% cholesterol, were purchased from Trophic Diets (Nantong, China). Oil-red O and Compound C were from Sigma (Missouri, United States). OxLDL, dioctadecyl-3,3,3,3-tetramethylin docarbocyanine-oxLDL (Dil-oxLDL) and high density lipoprotein (HDL) were from Yiyuan Biotech (Guangzhou, China). Apolipoprotein A1 (ApoA1) was from Novoprotein (Suzhou, China). 22-(N(-7-nitrobenz-2-oxa-1,3-diazol-4-yl)amino)-23,24-bisnor-5-cholen-3β-ol (NBD-cholesterol) and 4,4’-difluoro-4-bora-3a,4a-diaza-s-indacene (BODIPY) were from Thermo Fisher Scientific (Massachusetts, United States). Control, LOX-1 siRNA and SIRT6 siRNA were synthesized by Ribobio (Guangzhou, China). The sequences for LOX-1 siRNA were 5’-GCGUUUCUUUACAGCUAUATT -3’ and 5’-UAUAGCUGUAAAGAAACGCTT-3’, SIRT6 siRNA were 5’-GUGCAUGUUUCGUAUAAGUTT-3’ and 5’-ACUUAUACGAAACAUGCACTT-3’, and control siRNA were 5’-UUCUCCGAACGUGUCACGUTT-3’ and 5’-ACGUGACACGUUCGGAGAATT-3’.

### Human samples

Human atherosclerotic plaques from carotid arteries were obtained from patients who underwent carotid endarterectomy (CEA) procedure in The First Affiliated Hospital of Xi’an Jiaotong University, control carotid arteries were from age-matched donors. All procedures involving human samples were approved by the Research Ethics Committees of The First Affiliated Hospital of Xi’an Jiaotong University and conformed to the principles outlined in the Declaration of Helsinki. All relevant ethical regulations were followed in this study. Informed consent was obtained from all participants.

### Mice

Male apolipoprotein E-deficient (ApoE^−/−^) mice at 8 weeks old were from Vital River (Beijing, China) and randomly divided into two groups for 12-week high-fat diet (HFD) or normal chow diet (ND) feeding by using a random number table. Briefly, before grouping, each mouse was allocated one of the consecutive random numbers from the random number table in order of body weight. Then, all the mice were ranged by their random numbers from small to large and every consecutive 6 mice were assigned as one group.

Smooth muscle-specific LKB1 knockout (LKB1^SMKO^) mice were generated as described previously [30]. In brief, LKB1^flox/flox^ mice were hybridized with SM22α-CreER^T2^ transgenic mice expressing a tamoxifen-inducible Cre recombinase under the control of the SM22α promoter. Six-week-old LKB1^flox/flox^/Cre+ mice received continuous intraperitoneal injections of tamoxifen (1 mg/day) for 5 days to obtain LKB1^SMKO^ mice. Littermate LKB1^flox/flox^/Cre- mice receiving the same dose of tamoxifen were used as controls (CTR). Eight-week-old male control and LKB1^SMKO^ mice were subjected to a single tail-vein injection with AAV8/D377Y-mPCSK9 at 1.5 × 10^11 ^viral genomes (v.g.) for each mouse as described [70] and fed a Paigen diet for 12 weeks.

To knockdown LOX-1 in smooth muscle, 7-week-old male control/LKB1^SMKO^ mice were randomly divided into two groups by using a random number table as described above and injected with AAV9-shCON or AAV9-shLOX-1 at 1.5 × 10^11 ^v.g. via the tail vein. One week later, all the mice were subjected to a tail-vein injection of AAV8/D377Y-mPCSK9 at 1.5 × 10^11^ v.g. and 12-week Paigen diet feeding.

We set the significance level (α) at 0.05, and power (1-β) at 80% to determine the sample size according to our preliminary experiments. No samples or animals were excluded from data analysis. The exact number of groups is included in figure legends. All animals were housed under a 12-h day/night cycle at 25 °C. All experiments involving animals were conducted in accordance with the protocols approved by the Animal Care and Use Committee of Shandong University. All procedures conform to the National Institutes of Health (NIH) Guide for the Care and Use of Laboratory Animals. Investigators were blinded to the allocation of different groups when performing outcome evaluations.

### Cell culture

Vascular smooth muscle cells (VSMCs) were extracted from mouse aortas. Eight-week-old mice were euthanized with an intraperitoneal injection of sodium pentobarbital (100 mg/kg). Their aortas were excised and transferred to culture dishes containing phosphate-buffered saline (PBS). After dissection of the adventitia, tunica media tissues were cut into pieces and applied evenly to the bottom of a culture flask, which was then placed in an incubator at 37 °C and 5% CO_2_ for 2 h. After that, the culture flask was turned over and supplied with Dulbecco’s Modified Eagle Medium (DMEM) containing 15% fetal bovine serum, 100 μg/ml streptomycin, and 100 U/mL penicillin in the incubator for 5–7 days. Then, the fetal bovine serum in the culture medium for VSMCs was reduced to 10%.

HEK 293 T cells were purchased from American Type Culture Collection (Maryland, USA), and cultured in DMEM containing 10% fetal bovine serum, 100 μg/ml streptomycin, and 100 U/mL penicillin.

### Atherosclerotic lesion assay

The mice were euthanized as stated above and perfused with saline. The hearts and aortas (from the proximal ascending aorta to the common iliac artery bifurcation) were excised and fixed in 4% paraformaldehyde for 24 h. After the adventitia was stripped, the aortas were opened longitudinally and stained with Oil-red O for 30 min for aortic *en face* analysis. Hearts were embedded in optical cutting temperature (OCT) and sliced into 5μm-thick frozen sections from the region of the aortic root to reveal the aortic valves. The lesion area in the aortic root was quantified using hematoxylin and eosin (H&E) staining. Neutral lipid deposition was determined using Oil-red O staining.

### Immunofluorescence staining

Aortas were embedded in paraffin and then sectioned at a thickness of 5 μm. The sections were dewaxed and rehydrated before immunofluorescence staining. VSMCs were then incubated on round coverslips in 24-well plates. When cells grew to 50% of the total area of the coverslip, they were fixed in 4% paraformaldehyde for 10 min and then rinsed with PBS 3 times before immunofluorescence staining. Aortic sections or VSMCs coverslips were blocked with 5% normal goat serum in PBS for 30 min at room temperature and incubated overnight with primary antibodies at 4 °C. To detect the LKB1 expression level in VSMCs of aortas, fluorescent double labeling of aortic sections was performed with anti-LKB1 antibody (1:100, sc-32245, Santa Cruz) and anti-α-SMA antibody (1:200, ab5694, Abcam). To evaluate the co-localization of LKB1 and SIRT6, fluorescent double labeling of VSMCs coverslips was performed using anti-LKB1 and anti-SIRT6 antibodies (1:100, #12486, Cell Signaling Technology). After washing, sections or coverslips were incubated with secondary antibodies at 37 °C for 30 min and then stained with Mounting Medium with DAPI (ab104139, Abcam).

### Immunohistochemical analyses

Paraffin sections of aortas were dewaxed, rehydrated and then immunostained with anti-LOX-1 (1:100, ab60178, Abcam) to detect the expression of LOX-1. Frozen sections of the aortic roots were hydrated and immunostained with anti-MOMA-2 antibody (1:200, ab33451, Abcam) to determine the macrophage content.

### Lipid profile assays

After the mice were sacrificed, blood was collected and centrifuged to separate the serum. Serum levels of total cholesterol (TC), triglycerides (TG), high-density lipoprotein cholesterol (HDL-C), and low-density lipoprotein cholesterol (LDL-C) were measured using blood lipid assay kits from Jiancheng Bioengineering Institute (Nanjing, China).

### Oil-red O staining

VSMCs treated with oxLDL for 24 h were fixed with 4% paraformaldehyde in PBS for 10 min. After three cycles of washing with PBS, the cells were stained with Oil-red O for 30 min and stained with hematoxylin for 2 min at room temperature.

### BODIPY staining

Lipid droplets were detected with BODIPY staining. In brief, aortic root sections were dyed with 10 μg/mL BODIPY for 30 min, followed by immunofluorescence stained with anti-α-SMA antibody.

### Cholesterol uptake assay

Dil-oxLDL was used to determine cholesterol uptake. VSMCs were treated with 10 μg/mL Dil-oxLDL for 4 h, washed with PBS, and fixed in 4% paraformaldehyde in PBS. The uptake of Dil-oxLDL was analyzed by immunofluorescence.

### Cholesterol efflux assay

The VSMCs were incubated with NBD cholesterol for 4 h. After cholesterol loading, the cells were washed, equilibrated in a medium for 2 h, and then incubated with 20 μg/mL ApoA1 or 50 μg/mL HDL for 4 h. Control wells were treated with only 0.2% bovine serum albumin (BSA) to measure the background. The cell supernatant was collected, the cells were lysed at 37 °C with 0.3 M NaOH solution for 15 min, and the cell lysate was collected. The fluorescence intensities of the cell lysate and supernatant were measured using a microplate spectrophotometer. The efflux rate was calculated as follows: cholesterol efflux (%) = cell supernatant count/(cell supernatant count + cell lysate count) × 100.

### Western blot analysis

Proteins were extracted from cells on ice using RIPA buffer (P0013B, Beyotime) containing a protease/phosphatase inhibitor cocktail (Roche), separated by SDS-PAGE, and transferred to PVDF membranes. The membranes were blocked in 5% nonfat dry milk/Tween 20-tris buffered saline (TBST) for 1 h and incubated overnight at 4 °C with corresponding primary antibodies as follows: anti-LKB1 (1:1000, #3047, Cell Signaling Technology), anti-LOX-1 (1:1000, ab60178, Abcam), anti-SR-A1 (1:1000, ab151707, Abcam), anti-CD36 (1:1000, ab133625, Abcam), anti-SR-B1 (1:2000, ab52629, Abcam), anti-ABCA1(1:1000, #96292, Cell Signaling Technology), anti-ABCG1 (1:1000, ab52617, Abcam), anti-SIRT6 (1:1000, #12486, Cell Signaling Technology), anti-p-AMPKα (1:1000, #2535, Cell Signaling Technology), anti-AMPKα (1:1000, #5831, Cell Signaling Technology), anti-Phospho-(Ser/Thr) (1:1000, ab117253, Abcam), anti-GAPDH (1:1000, #5174, Cell Signaling Technology), and anti-Tubulin (1:1000, 11224-1-AP, Proteintech). After three washes with TBST, the membranes were incubated with appropriate horseradish peroxidase (HRP) conjugated secondary antibodies. After washing, the membranes were added dropwise to immobilized ECL ultra-western HRP substrate (Millipore) and imaged using a luminescent image analyzer (Amersham Imager 800, GE).

### Quantitative real-time PCR (qPCR)

According to the instructions, total RNA was extracted from VSMCs using an RNAfast2000 Total RNA Extraction Kit (220011, Fastagen). Total RNA was then reverse-transcribed into complementary DNA by using a PrimeScript^TM^ RT reagent Kit with gDNA Eraser (RR047A, Takara). PCR amplification was conducted using the TB Green^®^ *Premix Ex Taq*™ (RR420A, Takara). The oligonucleotide primer sequences were shown in Table S1.

### Chromatin immunoprecipitation (ChIP) assay and methylated DNA immunoprecipitation (MeDIP) assay

The ChIP assays were performed by SimpleChIP^®^ Enzymatic Chromatin IP Kit (#9003, Cell Signaling Technology) according to the manufacturer’s instructions. Briefly, after fixing, lysing, and sonicating the cells to prepare fragments, anti-methyl-CpG-binding protein 2 (MeCP2) (#3456, Cell Signaling Technology), anti-acetyl-histone H3 (Lys9) (H3K9ac) (ab4441, Abcam), and anti-acetyl-histone H3 (Lys56) (H3K56ac) (PA5-40101, Invitrogen) were incubated overnight to precipitate the chromatin. DNA was extracted, purified from the binding complex, and analyzed by qPCR. MeDIP assays were performed using a MeDIP kit (Bes5202, BersinBio) according to the manufacturer’s protocol. DNA was extracted from the cells, sonicated into fragments, and incubated overnight with anti-5-methylcytosine (5-MC) (ab214727, Abcam). After washing off the binding complex, DNA was eluted and analyzed by qPCR. Primers for LKB1 and LOX-1 promoters were shown in Table S1.

### Co-Immunoprecipitation (Co-IP)

VSMCs or HEK 293 T cells were lysed with IP lysis buffer (87787, Thermo Scientific) and incubated with anti-SIRT6 (#12486, Cell Signaling Technology), anti-HA (#3724, Cell Signaling Technology), anti-FLAG (#14793, Cell Signaling Technology), or IgG as a negative control at 4 °C overnight to form an antigen-antibody complex. Protein A/G magnetic beads (B23201, Bimake) were then added. After washing and magnetic separation, the precipitate was dissolved in 1 × SDS loading buffer for western blot analysis.

### Pull-down assay

GST or GST-SIRT6 (1 μg)-conjugated GSH beads were suspended in NETN buffer (20 mM Tris–HCl pH 8.0, 100 mM NaCl, 1 mM EDTA, 0.5% NP40 and phosphatase and protease inhibitor cocktail). The purified His-LKB1 proteins (1 μg) were then added and incubated overnight at 4 °C, followed by three washes with NETN buffer. The boiled eluates were then separated and detected with western blot analysis.

### SIRT6 activity assay

SIRT6 activity was measured using a fluorometric SIRT6 assay kit (ab156068, Abcam), following the manufacturer’s instructions. Briefly, VSMCs were transfected with SIRT6 siRNA to remove endogenous SIRT6 protein. After 48 h, VSMCs were transfected with SIRT6-Wild Type (WT), SIRT6-T51A, SIRT6-T57A, or SIRT6-T57A plasmids as well as adenovirus-expressing GFP or LKB1. The fluoro-substrate peptide, fluoro-deacetylated peptide, NAD, and developer were successively added to SIRT6 assay buffer, followed by incubation with the nuclear extract of VSMCs. Excitation was done at 480 nm and emitted light was detected at 530 nm using a microtiter plate fluorometer.

### Statistical analysis

All data were analyzed using GraphPad Prism 8.0 (GraphPad Software, USA) and presented as the mean ± SEM. The Shapiro–Wilk test was used to evaluate the Gaussian distribution of the data. Comparisons between two groups were conducted using the Student’s *t*-test, and comparisons between more than two groups were performed using one-way ANOVA with Bonferroni’s post-hoc test, as appropriate. Statistical significance was set at *P* < 0.05.

## Supplementary information

Online Supplemental Material

Original western blots with markers

checklist

## Data Availability

The data supporting the present study are available from the corresponding author upon reasonable request.
