# Supplementary material for: Smooth muscle liver kinase B1 inhibits foam cell formation and atherosclerosis via direct phosphorylation and activation of SIRT6
Source: Cell Death Dis. 2023 Aug 22;14(8):542. doi: 10.1038/s41419-023-06054-x (PMC10444762; doi:10.1038/s41419-023-06054-x)
Supplement: Supplementary file 1 — Online Supplemental Material [file 41419_2023_6054_MOESM1_ESM.docx]

**SUPPLEMENTAL MATERIAL**

**Smooth Muscle Liver Kinase B1 Inhibits Foam Cell Formation and Atherosclerosis via Direct Phosphorylation and Activation of SIRT6**

Qiming Deng^1^, Hongxuan Li^1^**^*^**, Xiaolin Yue^1^, Chenghu Guo^1^, Yuanyuan Sun^1^, Chang Ma^1^, Jiangang Gao^2^, Yue Wu^3^, Bin Du^3^, Jianmin Yang^1^, Cheng Zhang^1^**^*^**, Wencheng Zhang^1^**^*^**

^1^National Key Laboratory for Innovation and Transformation of Luobing Theory; The Key Laboratory of Cardiovascular Remodeling and Function Research, Chinese Ministry of Education, Chinese National Health Commission and Chinese Academy of Medical Sciences; Department of Cardiology, Qilu Hospital of Shandong University, Jinan, China; ^2^School of Life Science and Key Laboratory of the Ministry of Education for Experimental Teratology, Shandong University, Jinan, China; ^3^Department of Cardiology, The First Affiliated Hospital of Xi’an Jiaotong University, Xi’an, China.

***Correspondence to:** Wencheng Zhang, [zhangwencheng@sdu.edu.cn](mailto:zhangwencheng@sdu.edu.cn); Cheng Zhang, [zhangc@sdu.edu.cn](mailto:zhangc@sdu.edu.cn); Hongxuan Li, 1482063316@qq.com.

**Table S1 Primer sequences used in this study.**

| **Gene Name** | **Primer sequences for qPCR (5’-3’)** |
| --- | --- |
| Mouse LKB1 | Forward: CCGACAGATTAGGCAGCACA |
|  | Reverse: GGCTTGGTGGGATAGGTACG |
| Mouse GAPDH | Forward: TGTCTCCTGCGACTTCAACA |
|  | Reverse: GGTGGTCCAGGGTTTCTTACT |
| Mouse LKB1 CpG island 1 | Forward: CTGAGGGAGGCATTTCACTC |
| (for ChIP and MeDIP) | Reverse: GGCATAGAGAGGAGCGTTTC |
| Mouse LKB1 CpG island 2 | Forward: TTAGGGATCCACGAGCCTCTC |
| (for ChIP and MeDIP) | Reverse: CAGACGCCCGTCAGCAAG |
| Mouse LKB1 CpG island 3 | Forward: GTCGCTTGCTTACTCCCATTG |
| (for ChIP and MeDIP) | Reverse: GGCAGTTGGCTGTAGCTTTAG |
| Mouse LOX-1 | Forward: GTGTCATGGAGCATTCTGAATTT |
| (for ChIP) | Reverse: TAATCGTGGCTTGGGAACTCA |

**Table S2 Predicted LKB1 phosphorylation sites of SIRT6 by Group-based Prediction System (GPS, version 5.0) software.**

| **Species** | **Position** | **Code** | **Peptide** |
| --- | --- | --- | --- |
| Human | 51 | T | SSSVVFHTGAGISTA |
|  | 57 | T | HTGAGISTASGIPDF |
|  | 184 | T | CRGELRDTILDWEDS |
| Mouse | 51 | T | SSSVVFHTGAGISTA |
|  | 57 | T | HTGAGISTASGIPDF |
|  | 184 | T | CRGELRDTILDWEDS |
| Rat | 51 | T | SSSVVFHTGAGISTA |
|  | 57 | T | HTGAGISTASGIPDF |
|  | 184 | T | CRGELRDTILDWEDS |

**
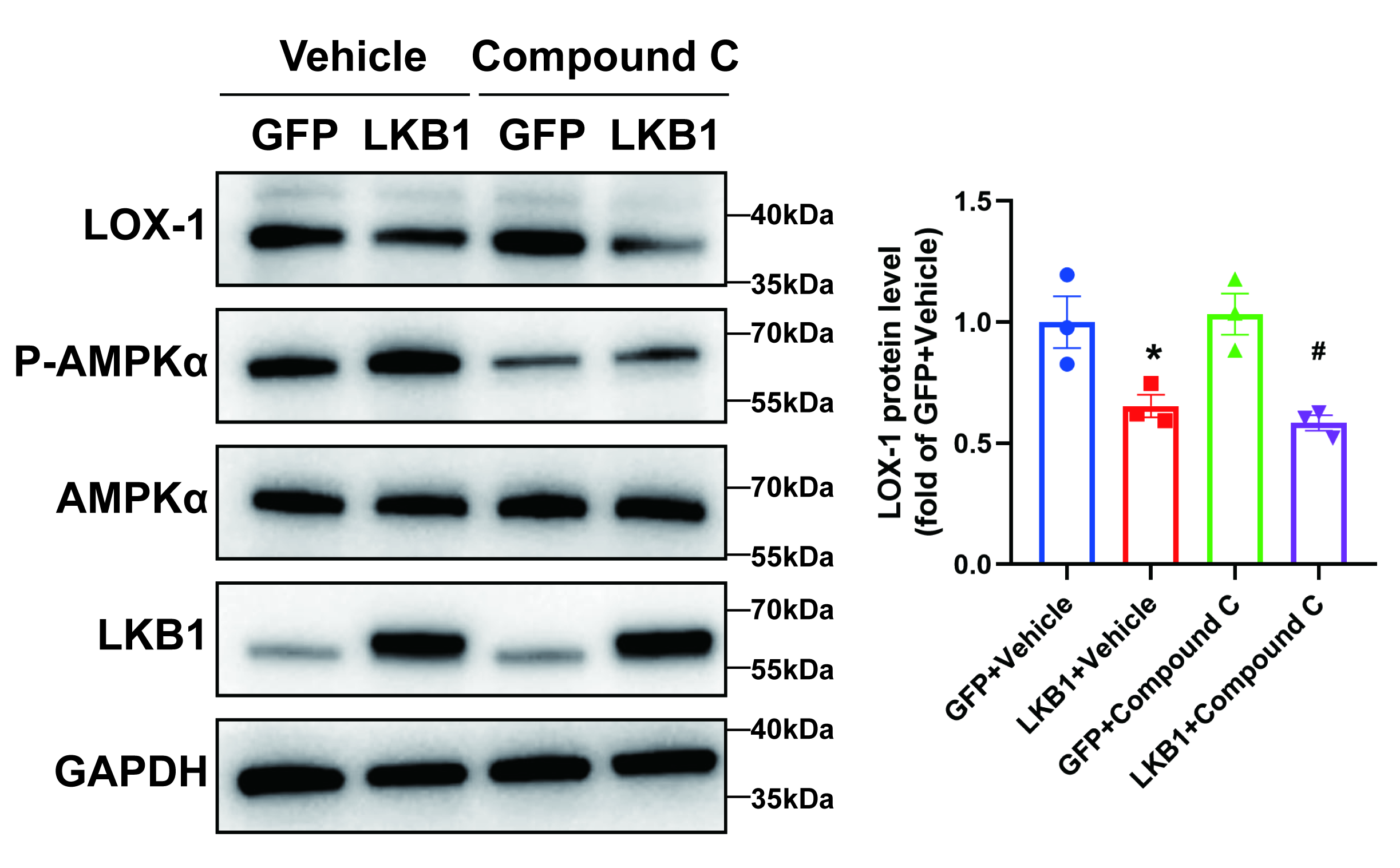
**

**Fig. S1 Inhibition of AMPK could not abolish the effect of LKB1 on downregulating LOX-1.** VSMCs were pretreated with Compound C (10 μM) 2 h before infection with adenovirus expressing GFP or LKB1. Western blot analysis for LOX-1 expression (n=3). ^*^P < 0.05 *vs* Vehicle+GFP, ^#^P < 0.05 *vs* Compound C+GFP. Data were analyzed by one-way ANOVA followed by Bonferroni multiple comparison analysis.


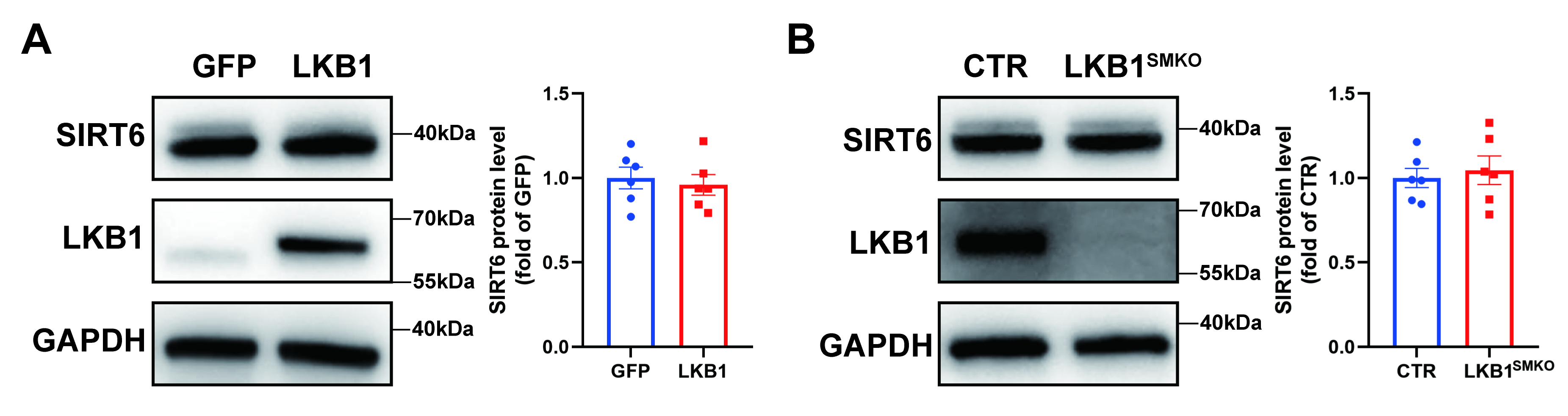


**Fig. S2 LKB1 does not affect the SIRT6 protein level.** **A** Western blot analysis for LOX-1 in VSMCs infected with adenovirus expressing GFP or LKB1 (n=6). **B** Western blot analysis for LOX-1 in VSMCs from CTR and LKB1^SMKO^ mice (n=6). Data were analyzed by two-tailed Student’s unpaired t-test (**A** and **B**).
