# Supplementary material for: Smooth muscle liver kinase B1 inhibits foam cell formation and atherosclerosis via direct phosphorylation and activation of SIRT6
Source: Cell Death Dis. 2023 Aug 22;14(8):542. doi: 10.1038/s41419-023-06054-x (PMC10444762; doi:10.1038/s41419-023-06054-x)
Supplement: Supplementary file 2 — Original western blots with markers [file 41419_2023_6054_MOESM2_ESM.pptx]

## Slide 1
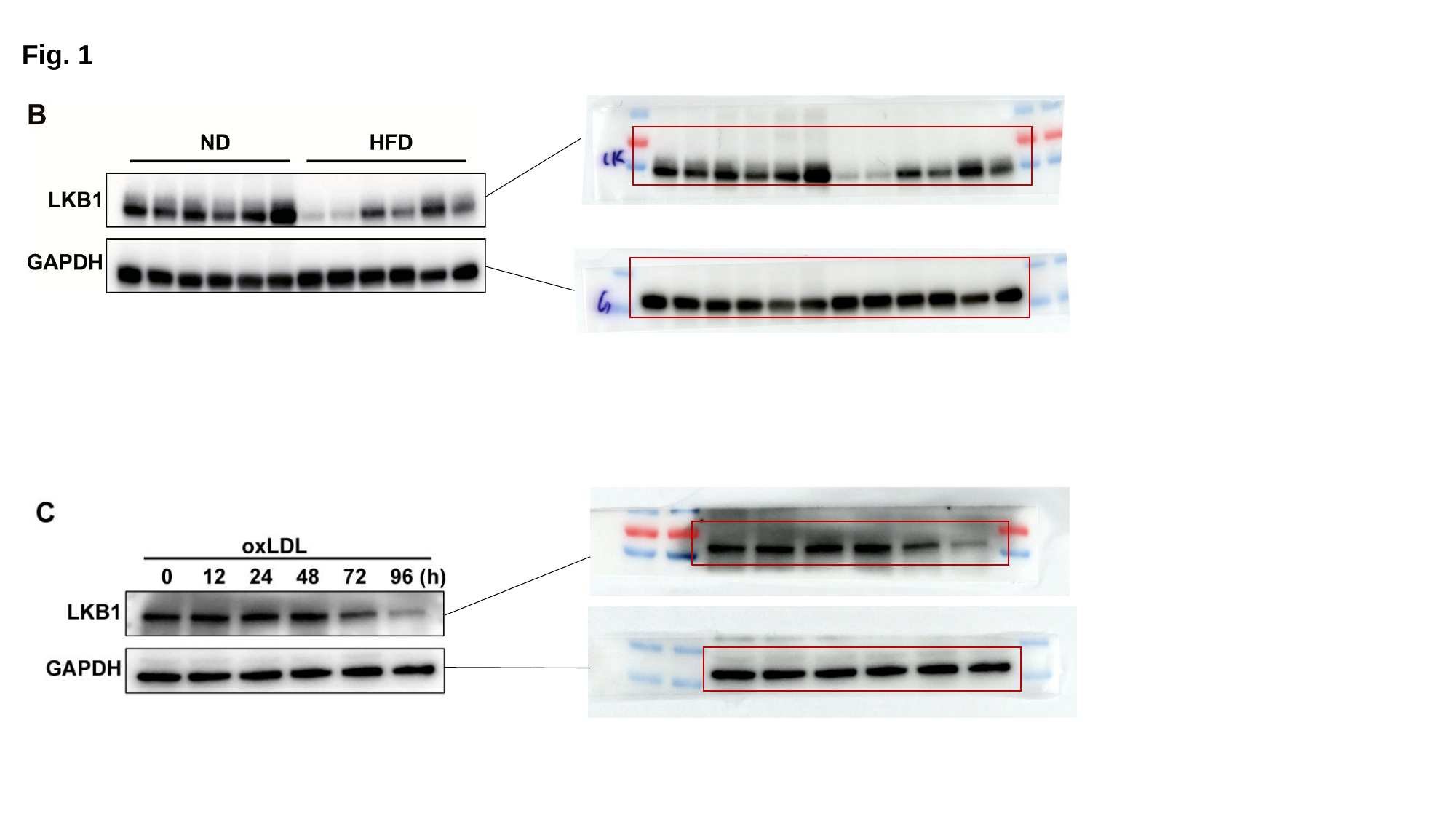

# Fig. 1

## Slide 2
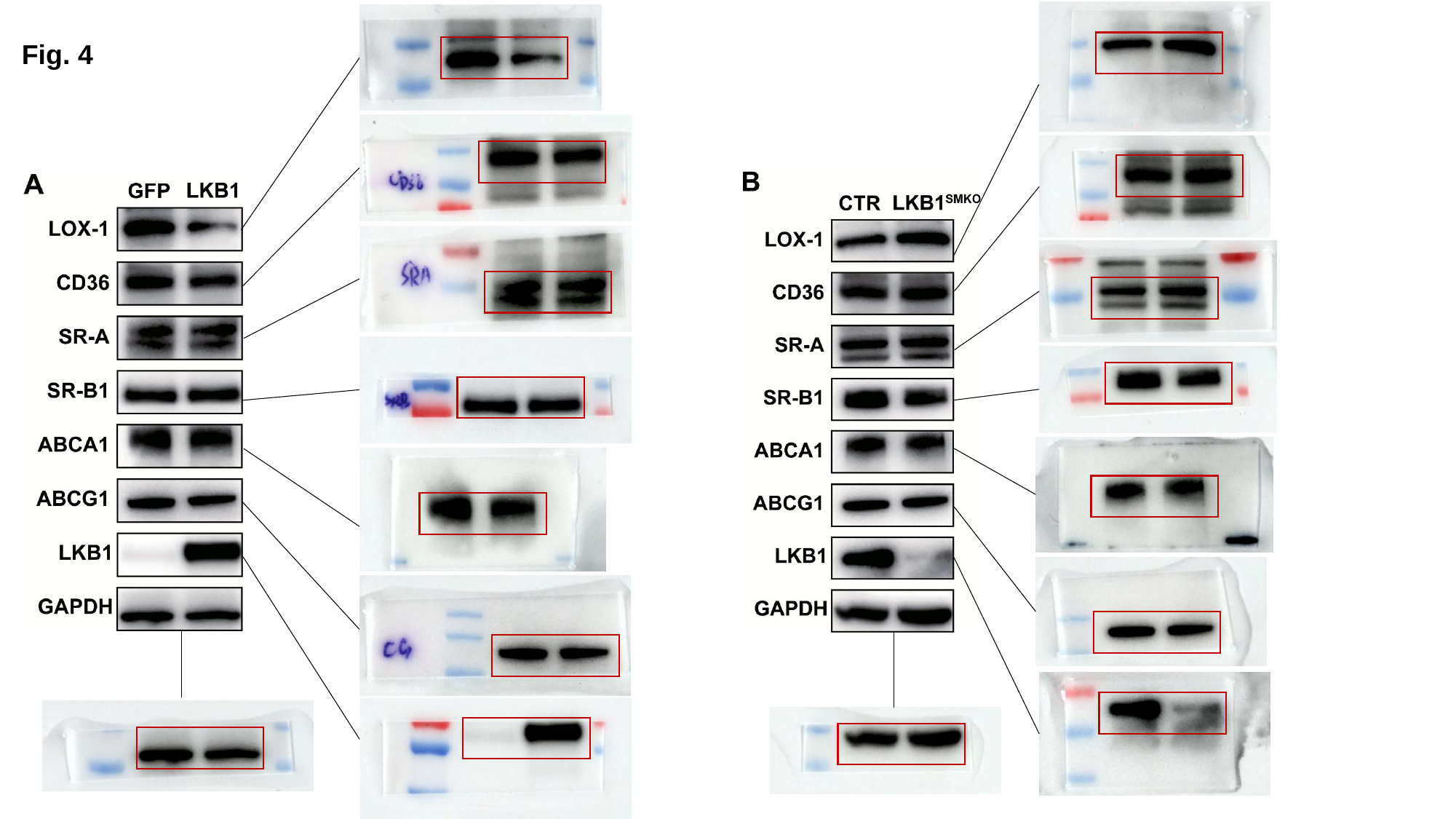

# Fig. 4

## Slide 3
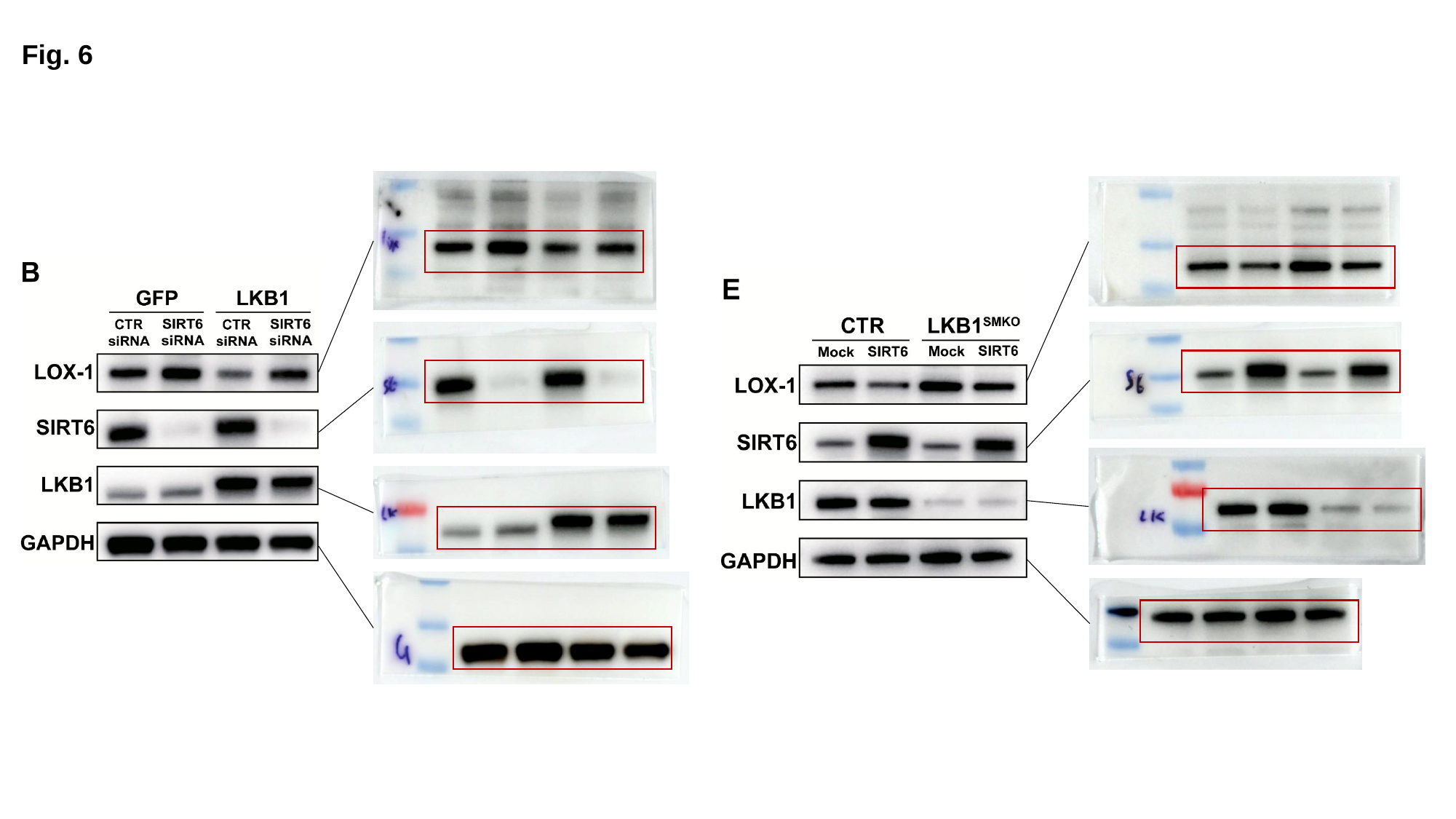

# Fig. 6

## Slide 4
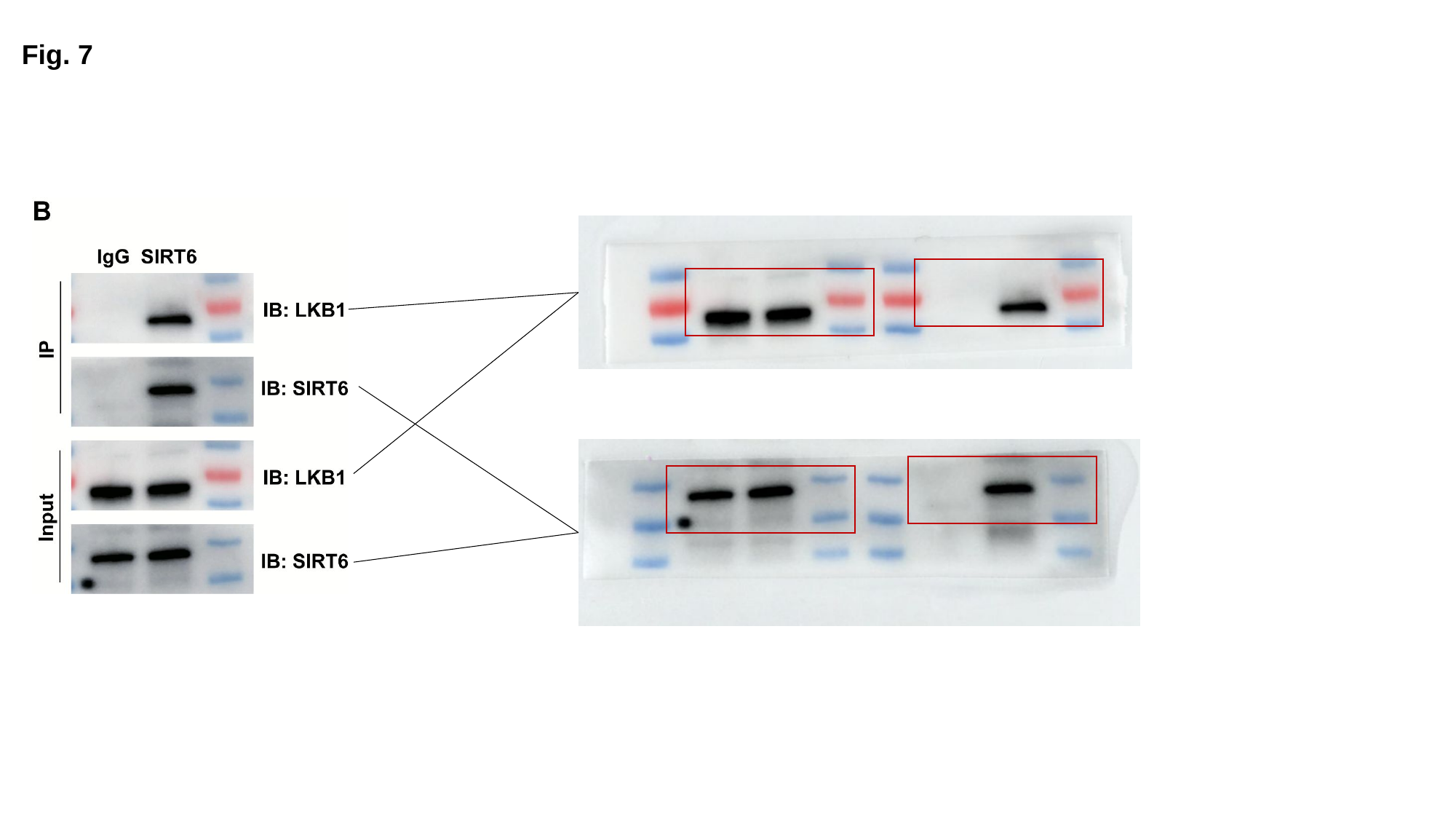

# Fig. 7

## Slide 5
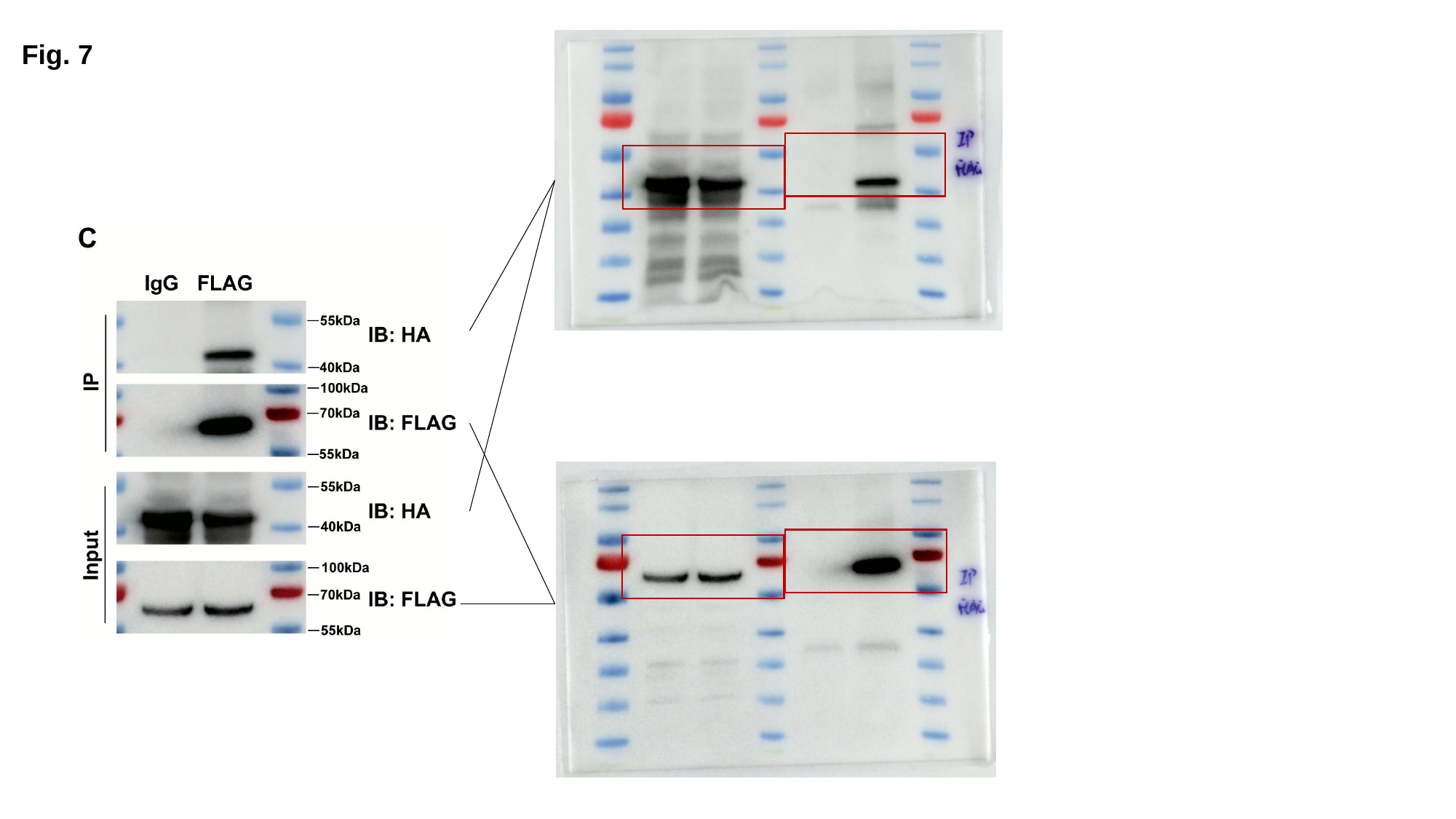

# Fig. 7

## Slide 6
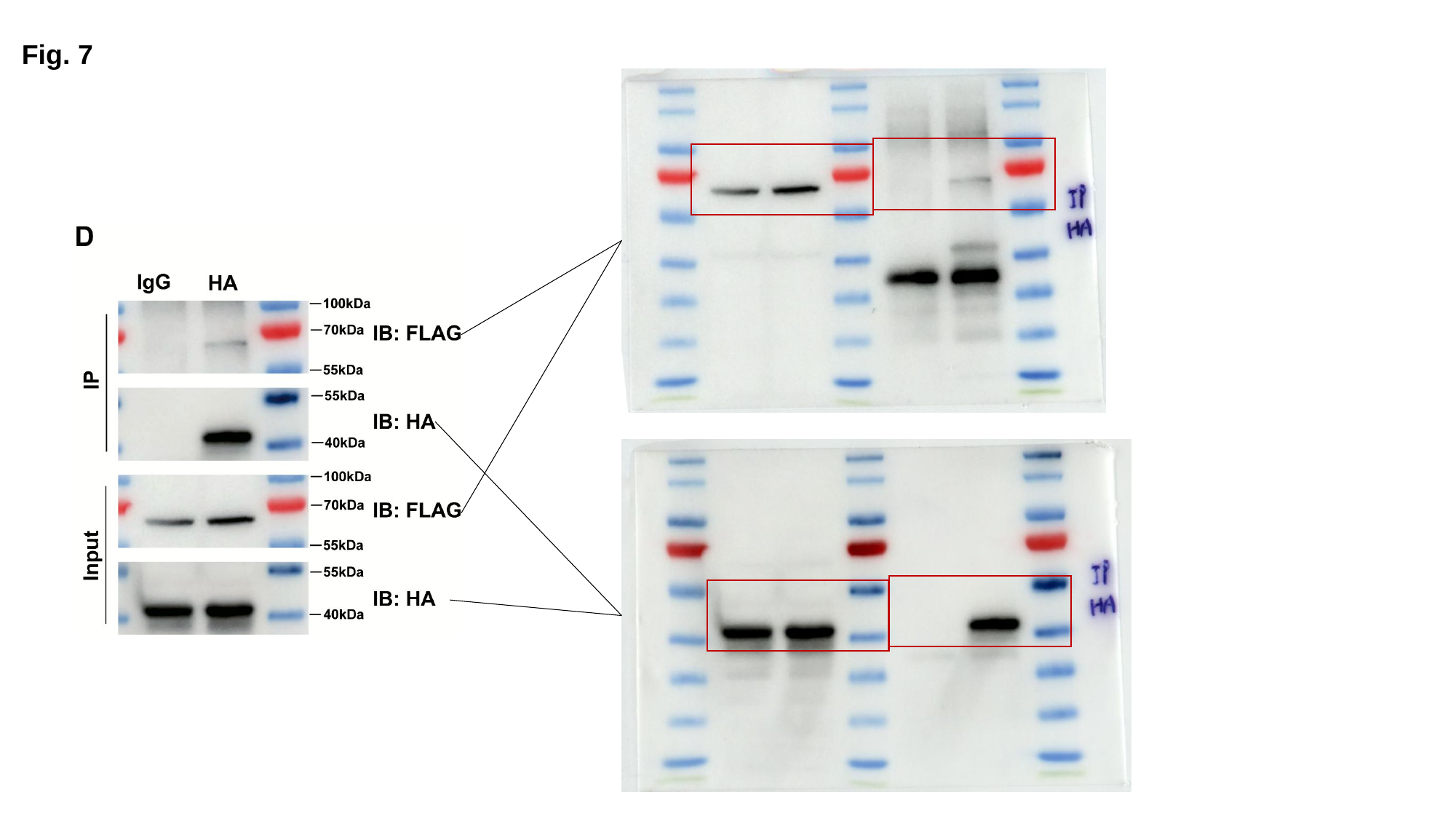

# Fig. 7

## Slide 7
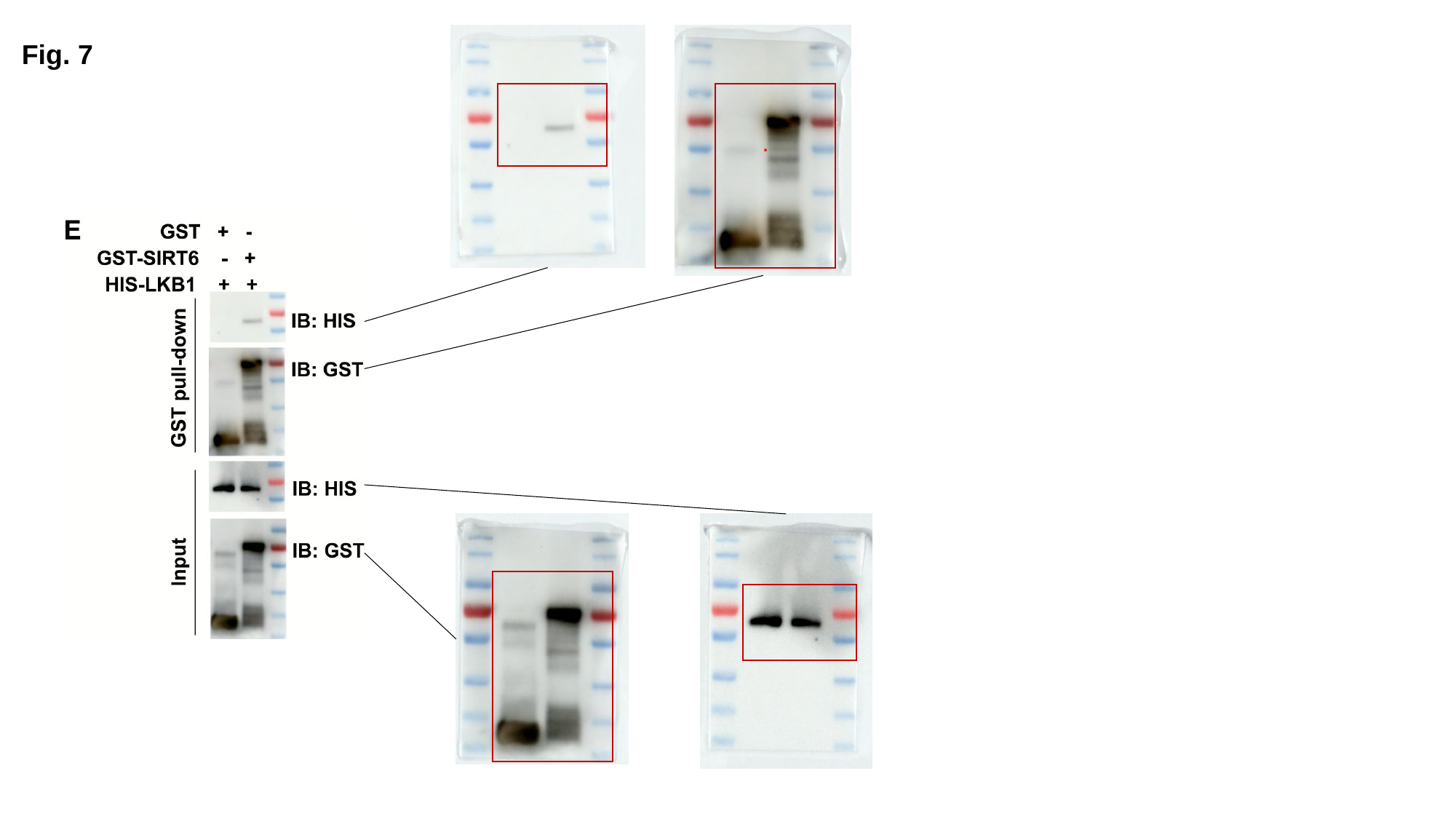

# Fig. 7

## Slide 8
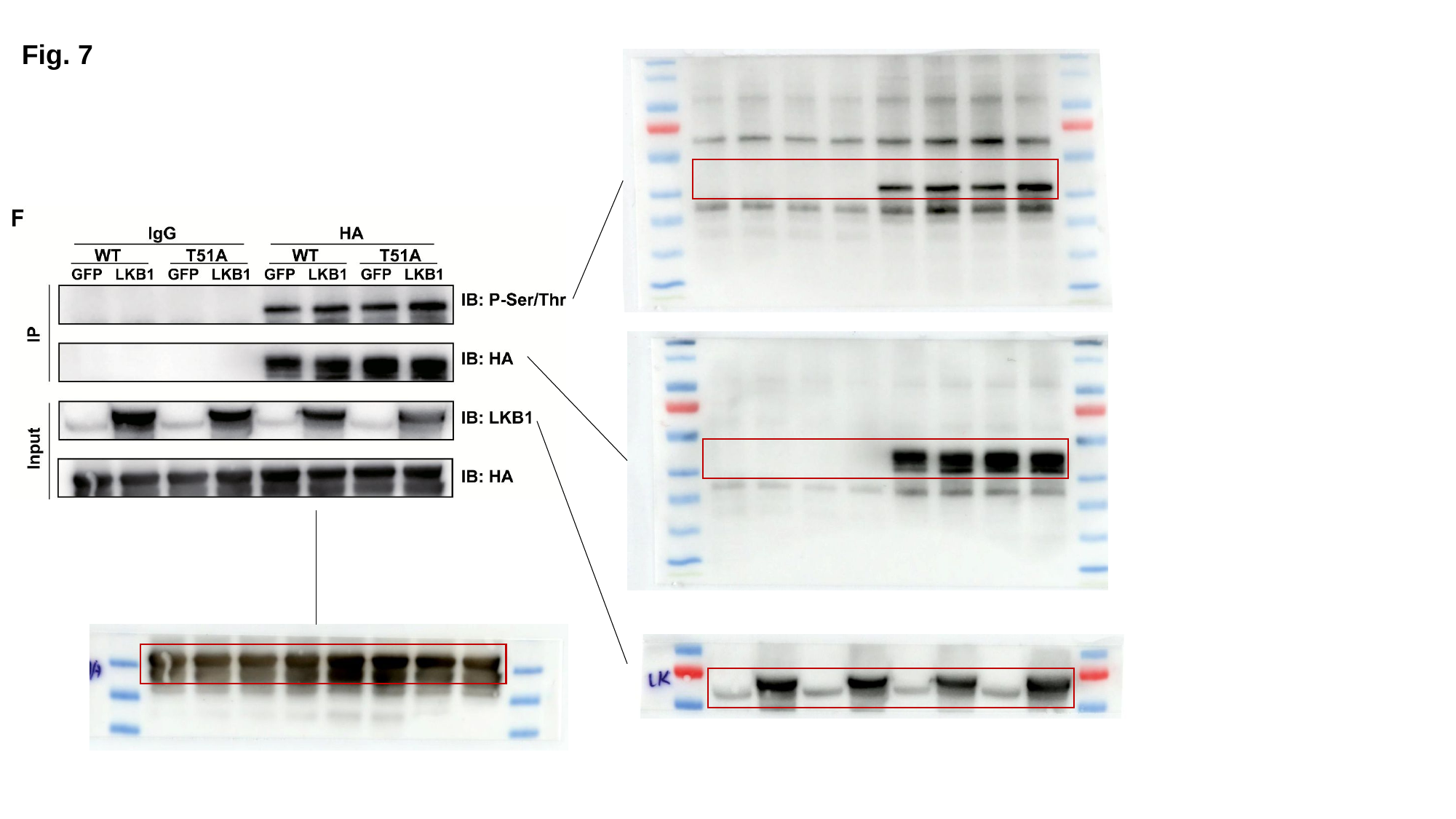

# Fig. 7

## Slide 9
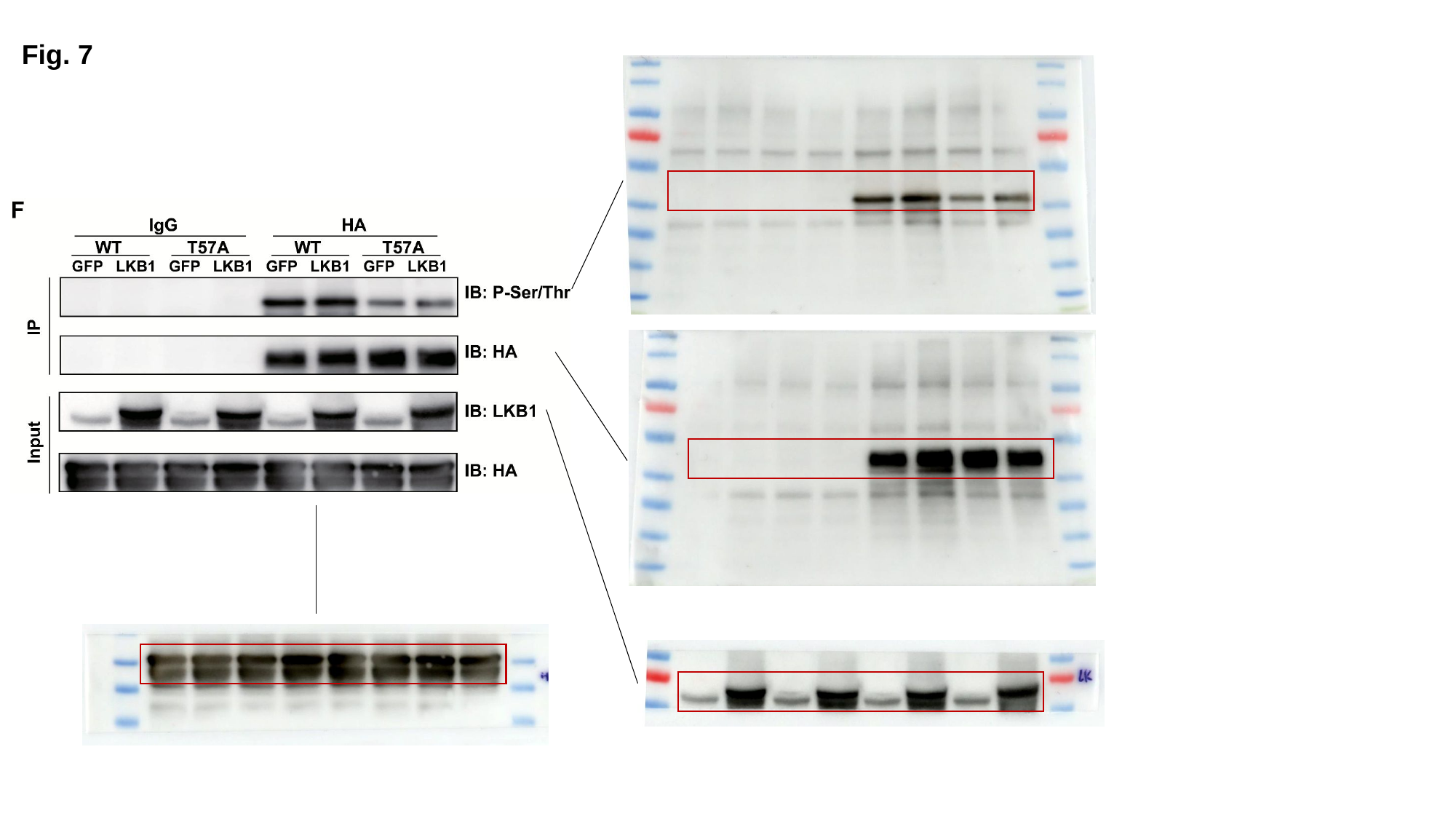

# Fig. 7

## Slide 10
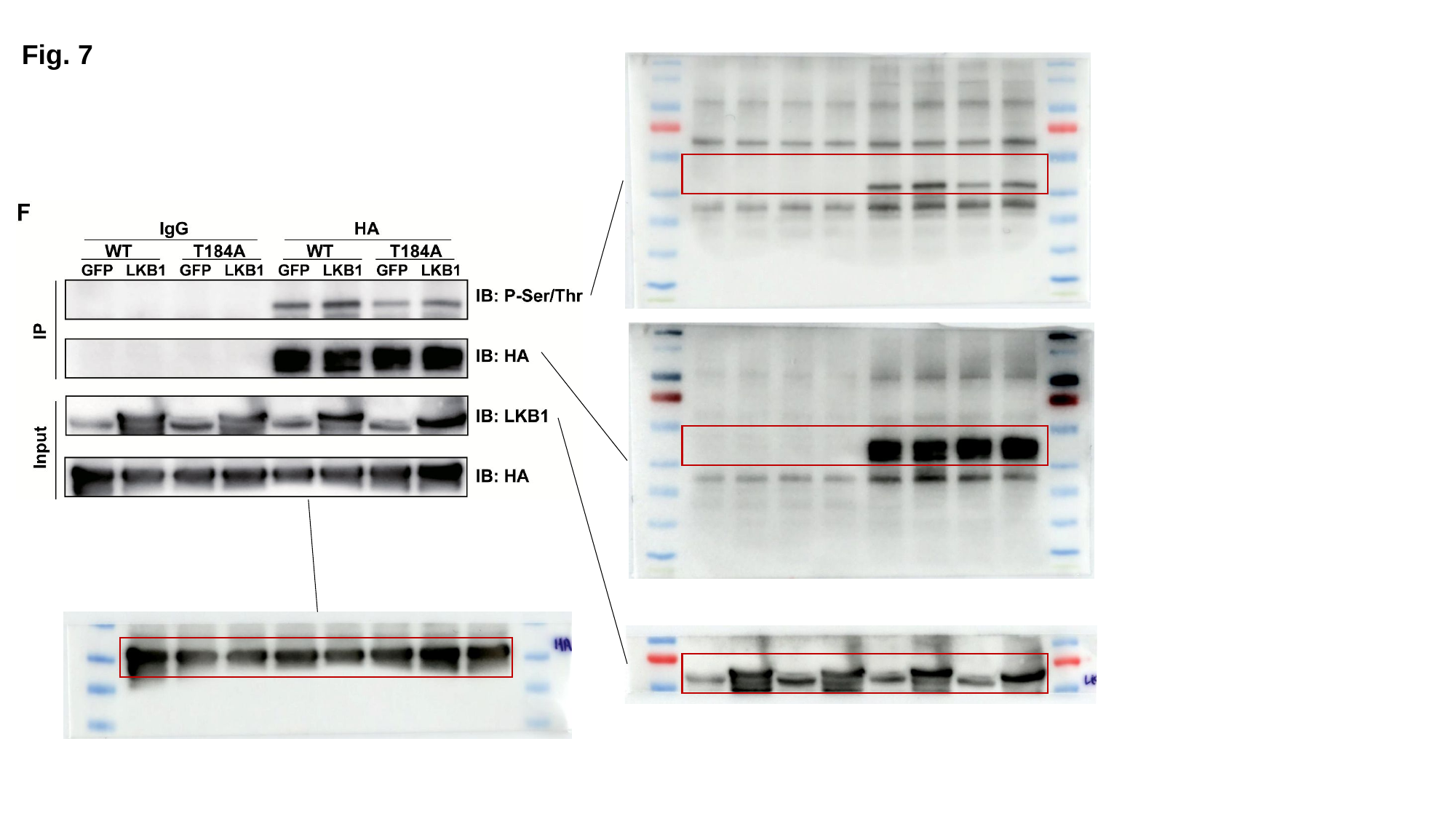

# Fig. 7

## Slide 11
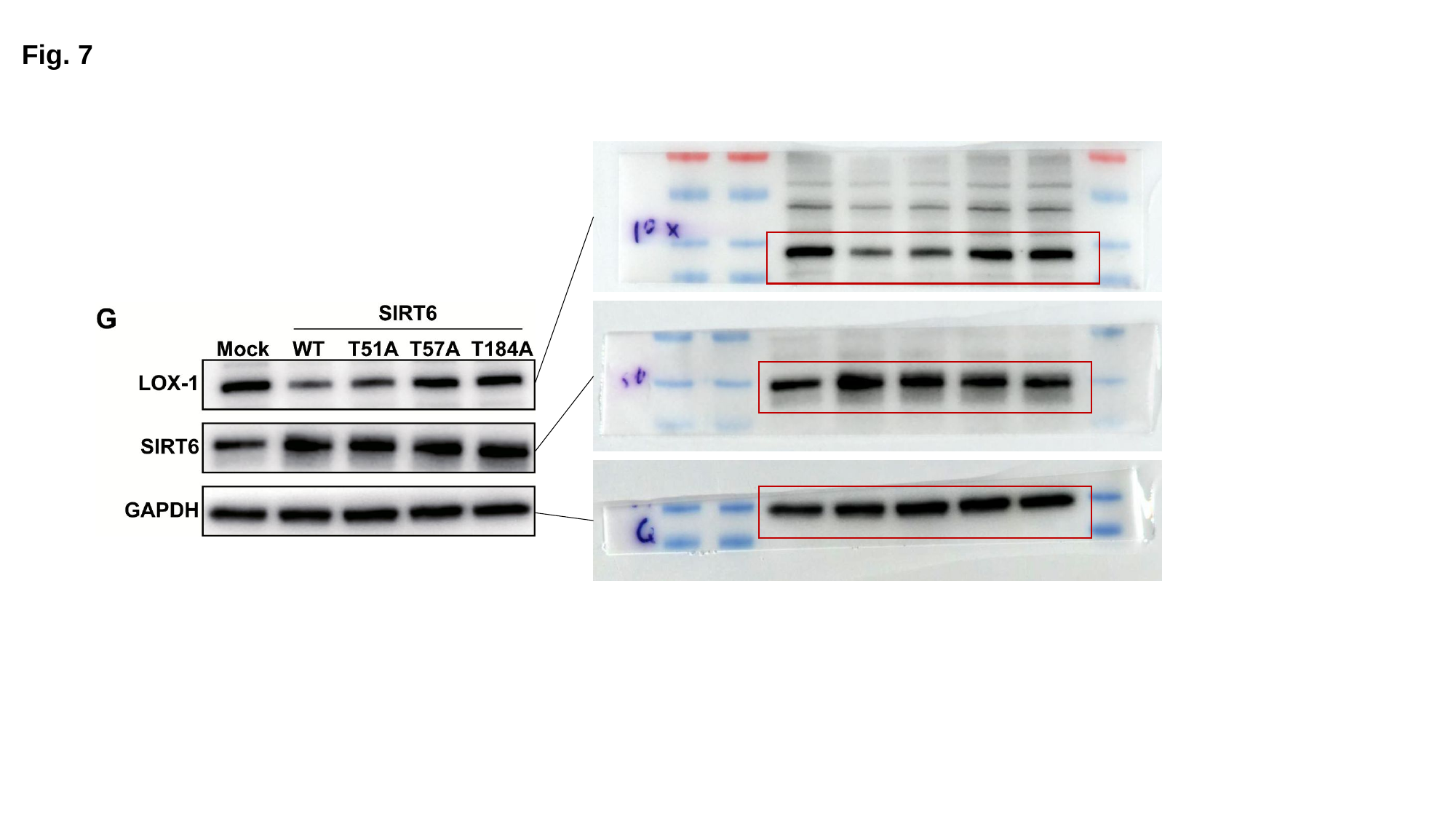

# Fig. 7

## Slide 12
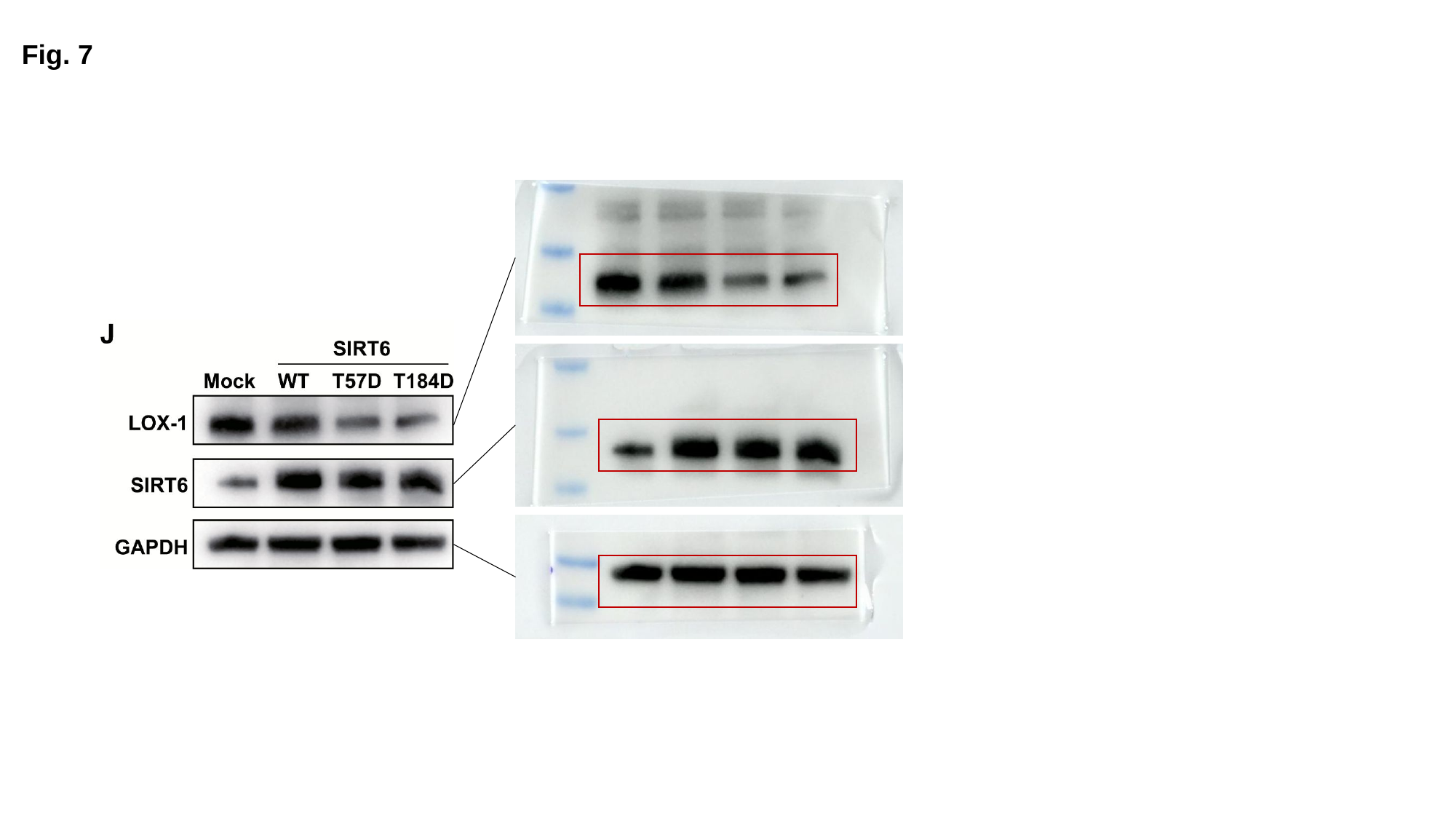

# Fig. 7

## Slide 13
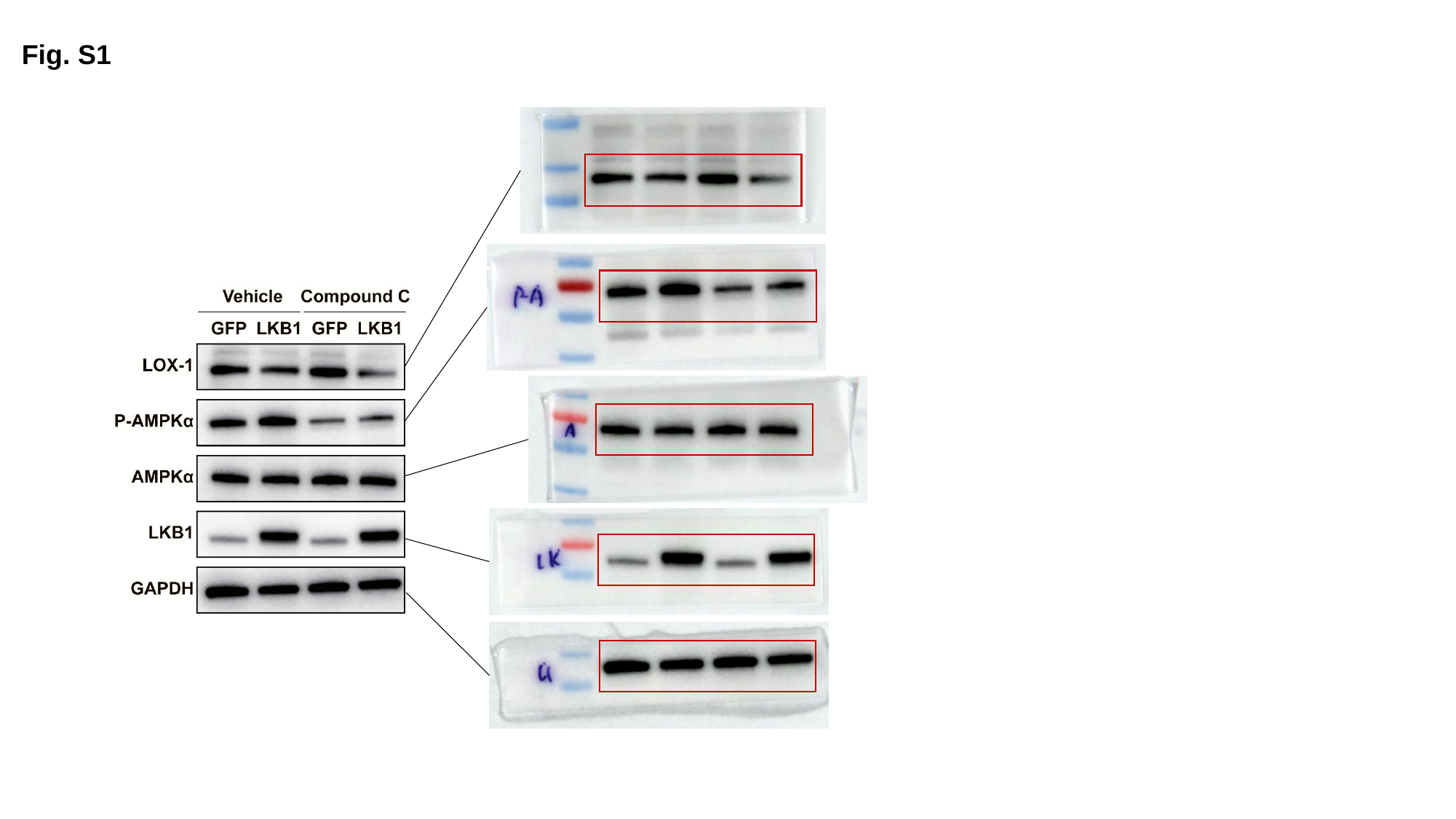

# Fig. S1

## Slide 14
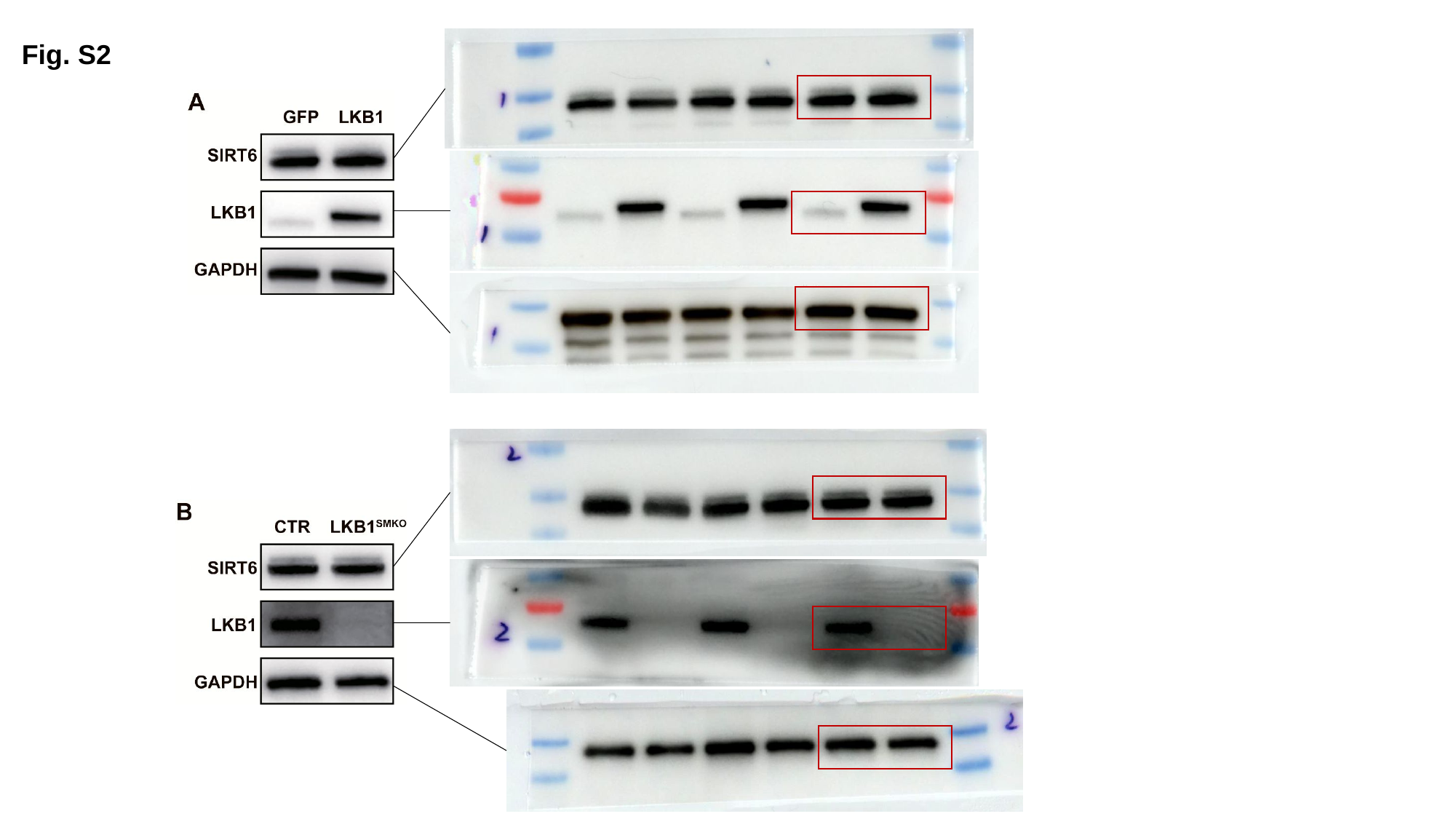

# Fig. S2
